# Proton irradiation: a key to the challenge of N-glycosidic bond formation in a prebiotic context

**DOI:** 10.1038/s41598-017-15392-8

**Published:** 2017-11-07

**Authors:** Raffaele Saladino, Bruno M. Bizzarri, Lorenzo Botta, Jiří Šponer, Judit E. Šponer, Thomas Georgelin, Maguy Jaber, Baptiste Rigaud, Mikhail Kapralov, Gennady N. Timoshenko, Alexei Rozanov, Eugene Krasavin, Anna Maria Timperio, Ernesto Di Mauro

**Affiliations:** 1 Department of Ecological and Biological Sciences, Via S. Camillo de Lellis, University of Tuscia, 01100 Viterbo, Italy; 2 Institute of Biophysics, Academy of Sciences of the Czech Republic, Královopolská 135, CZ-61265 Brno, Czech Republic; 3 Regional Centre of Advanced Technologies and Materials, Department of Physical Chemistry, Faculty of Science, Palacky University, 17. Listopadu, 771 46 Olomouc, Czech Republic; 4 Sorbonne Universités, UPMC Paris 06, CNRS UMR 7197, Laboratoire de Réactivité de Surface 4 place Jussieu, F-75005 Paris, France; 5 Centre de Biophysique Moleculaire, UPR CNRS4301 Orléans, France; 6 Sorbonne Universités, UPMC Paris06, CNRS UMR 8220, Laboratoire d’Archéologie Moléculaire et Structurale, Paris, France; 7 CNRS Institut des Matériaux de Paris Centre (FR2482), Paris, France; 8 Joint Institute for Nuclear Research, JINR’s Laboratory of Radiation Biology, Dubna, Russia

## Abstract

The formation of nucleosides in abiotic conditions is a major hurdle in origin-of-life studies. We have determined the pathway of a general reaction leading to the one-pot synthesis of ribo- and 2′-deoxy-ribonucleosides from sugars and purine nucleobases under proton irradiation in the presence of a chondrite meteorite. These conditions simulate the presumptive conditions in space or on an early Earth fluxed by slow protons from the solar wind, potentially mimicking a plausible prebiotic scenario. The reaction (i) requires neither pre-activated precursors nor intermediate purification/concentration steps, (ii) is based on a defined radical mechanism, and (iii) is characterized by stereoselectivity, regioselectivity and (poly)glycosylation. The yield is enhanced by formamide and meteorite relative to the control reaction.

## Introduction

In the consensus “RNA world” scenario framing the Origin of Life, the generation of nucleosides in abiotic conditions remains the major initial hurdle. The one-pot formation of nucleosides from formamide NH_2_CHO was reported^1^ but without a detailed mechanistic insight into the sequence of contributing chemical events. The experimental set-up consisted of formamide, of meteorites as catalysts, and of a 170 MeV proton beam as energy source modelling the Solar Wind radiation. In these conditions the formation of cytidine, uridine, adenosine, and thymidine was observed without isolation and/or purification of specific intermediates, along with a variety of organic compounds including nucleobases (cytosine, uracil, adenine, guanine, and thymine), carbohydrates (noticeably, ribose and 2-deoxy-ribose), aminoacids and carboxylic acids.

Formamide is among others the most intensively studied chemical precursor for prebiotic syntheses^2–5^. It has been detected in dense diffuse clouds in interstellar space in the galactic habitable zone^6^, in comets^7^ and satellites^8^. For the prebiotic relevance of formamide see SI # 1.

Nucleosides are made of two components: heterocyclic nucleobases and carbohydrates. Nucleobases are easily synthesized under prebiotic conditions^2,3,9^ and have marked stability. Carbohydrates are more problematic, both for their synthesis and for their stability^8–10^. Still, the prebiotic synthesis of numerous carbohydrates was recently reported^1,11–13^ including, among others, ribose and 2-deoxyribose^13^.

Due to the hurdles surrounding the formation of β-glycosidic bonds between nucleobases and sugars (for a recent review see^14^) plausibility of nucleosides in a prebiotic context has been disputed. This motivated recent efforts aimed at elaborating chemical routes to nucleosides which do not involve the β-glycosylation step. It has recently been reported that pyrimidine ribonucleosides are afforded by the oxazoline chemistry^15^, recently revisited^16,17^, encompassig formamide as solvent for the thiolysis and phosphorylation of anhydronucleoside intermediates^18^. Alternatively, purine ribonucleosides are synthesized through the formamido pyrimidines (FPy) chemistry^19^, requiring formamide in the *N*-formylation of amino pyrimidine intermediates for the purine ring-closure. This reaction is the reverse version of the previously reported formation of FPy by treatment of purine nucleosides with formamide^20^, described in the cyclic conversion of purine into pyrimidine nucleobases^9^. Remarkably, formamide plays a role in both pathways.

Prebiotic oxazoline and formamido pyrimidine syntheses are both multi-steps procedures based on the construction of the heterocyclic nucleobase scaffold on a pre-formed and anomerically functionalized carbohydrate. The results obtained define two different, equally ingenuous and informative, routes to the ex novo synthesis of nucleosides. However, the repeatedly required interventions of an operator, which are necessary in both oxazoline- and formamido pyrimidine-based syntheses, justify caution about their prebiotic plausibility. This leads us to ask: does a simpler, more direct reaction pathway exists which allows for the one-pot synthesis of nucleosides via formation of the β-glycosidic bond between separately preformed sugar and nucleobase moieties? The same question was asked by Orgel and collaborators more than 40 years ago^15,21^.

Since nucleobases and carbohydrates are produced by independent reactions from formamide^22^, including proton irradiation conditions^1^, we have analyzed the possibility that the β-glycosidic bond might form directly between them under suitable prebiotic conditions, challenging the endergonic character of the reaction. Proton irradiation looked to be the appropriate choice for this purpose. Given the known induction of radical species by proton irradiation, the results are interpreted and discussed in the frame of radical chemistry.

## Results

Irradiation experiments of nucleobases and carbohydrates were performed in three experimental conditions: 1, carbohydrate and adenine in solid film; 2, carbohydrate and adenine in formamide; 3, carbohydrate and adenine in formamide in the presence of powdered NWA 1465, selected as representative chondrite meteorite. Inorganic and organic composition, and cosmo-origin data of NWA 1465 are in SI # 2.

The solid film of adenine and carbohydrate (2-deoxyribose or ribose), or their solution in formamide (2.5 mL), with or without NWA 1465 powder (1.0% in weight, corresponding to 28 mg), was irradiated at 243 K with 170 MeV protons for 3 min. The uniform proton field was bounded to 10 × 10 cm^2^ by the collimator system. The averaged linear energy transfer (LET) was 0.57 keV/μm and the calculated absorbed dose was 6 Gy. The presence of organics in the original sample of NWA 1465, observed in the ppb range^23^, was prevented by extracting the powder with NaOH (0.1 N), CHCl_3_-CH_3_OH (2:1 v/v) and sulfuric acid. The endogenous organics possibly present were removed by the extracting mixture during the treatment, and the remaining powder was used for the irradiation experiments. In agreement with previous data, the treated and untreated NWA 1465 powder performed as catalyst similarly during formamide irradiation (Table 1, note c)^13^. Materials and Methods are detailed in experimental methods and in SI # 3. The analysis was limited to products ≥1 ng/mL and the yield was calculated as percentage (%) of product per starting adenine.

**Table 1:** Reaction of adenine (**1**) with 2-deoxyribose (**2**).

Entry	Conditions	Adenine^a^ (%)	Product (%)^d^					
			α-dfA(3)	β-dfA (4)	α-dpA (5)	β-dpA (6)	df(p)A 7a	poly-df(p)A 7b
1	Dry state^b^	51	9.2	3.1	19.5	5.4	6.3	0.6
2	FA	20	16.3	8.1	32.8	9.9	9.8	1.4
3	FA + meteorite	≤1	21.3 (21.3)^c^	30.2 (30.1)	25.1 (25.2)	6.8 (6.8)	15.2 (15.1)	1.4 (1.4)

FA = NH_2_CHO. Meteorite = NWA 1465. The reaction provides furanosides (f) and pyranosides (p) as α- and β-isomers. **α-dfA** = α-D-2′-deoxy-ribofuranosyl adenine; **β-dfA** = β-D-2′-deoxy-ribofuranosyl adenine; **α-dpA** = α-D-2′-deoxy-ribopyranosyl adenine; **β-dpA** = β-D-2′-deoxy-ribopiranosyl adenine; **df(p)A** = N^6^-glycosyl-2′-deoxyadenosine isomers; **poly-df(p)A** = N^6,6^-bis-glycosylated-2′-deoxyadenosine isomers. ^a^Unreacted adenine. ^b^Obtained after dissolution of 2-deoxyribose in distilled water and successive drying under nitrogen. ^c^Data obtained in the reaction with untreated NWA 1465. ^d^The yield was calculated as percentage (%) of nucleoside (mmol) with respect to starting adenine. The data are the mean values of three experiments with standard deviation equal to or less than 0.1%.

We focused on the detection of nucleosides and nucleoside derivatives by ultrahigh performance liquid chromatography on-line to orbitrap-mass-spectrometry (UHPLC-MS/MS; Q Exactive-orbitrap mass analyzer). The structure of products was unambiguously defined by comparison of the mass fragmentation peaks with original commercial samples and with specifically laboratory-prepared samples as standards. The nucleosides showed all the expected molecular ions (M) and the specific fragment ions. When necessary, the assignment was further confirmed by the Addition Method^19^ and by Matrix-assisted laser desorption/ionization mass spectrometry (MALDI) TOF analysis.

**Figure 1:**
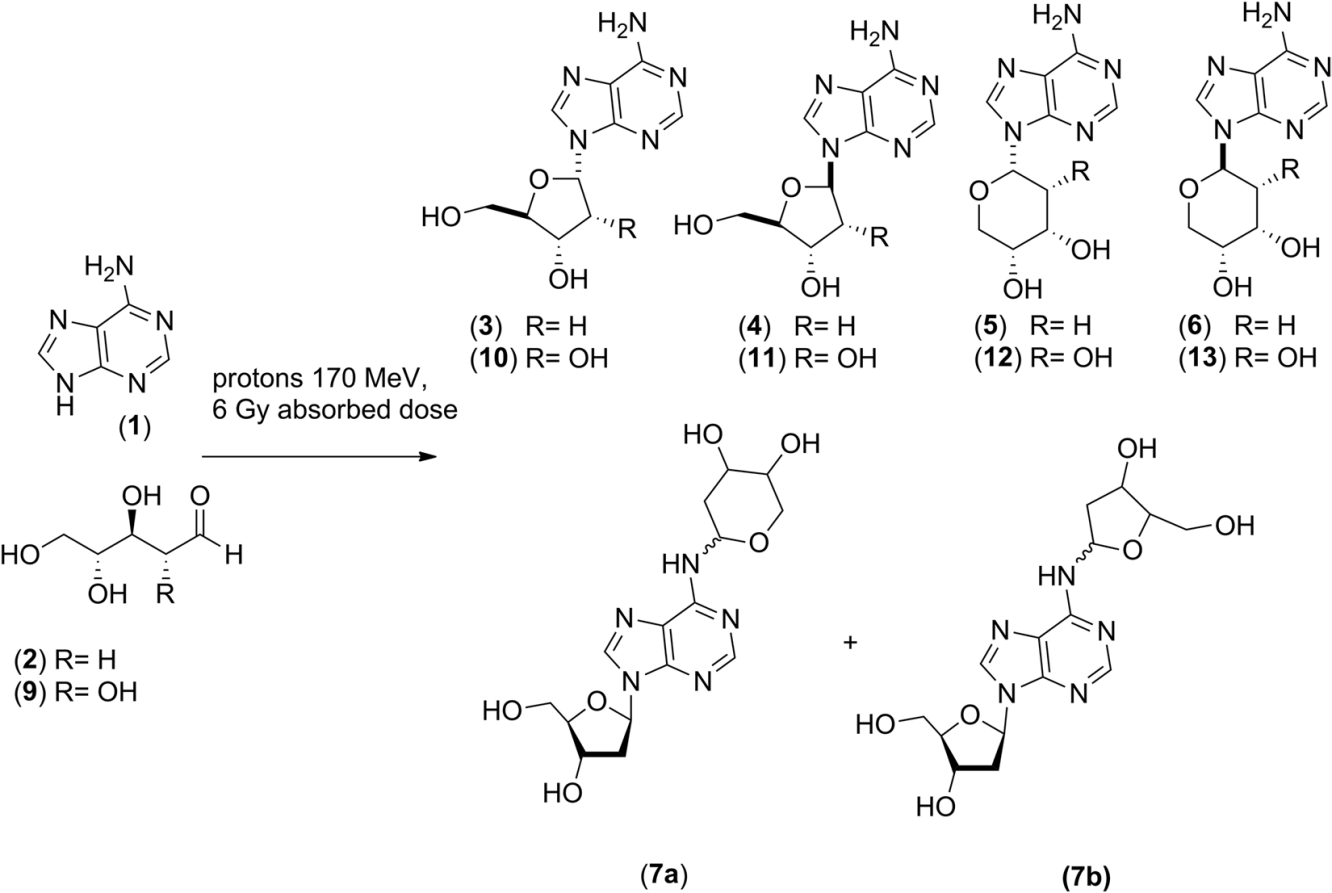
Formation of adenosine nucleosides by irradiation of adenine (**1**) and carbohydrates (**2**) and (**9**) (**2**: 2-deoxy-D-ribose. **9**: D-ribose).

**Figure 2:**
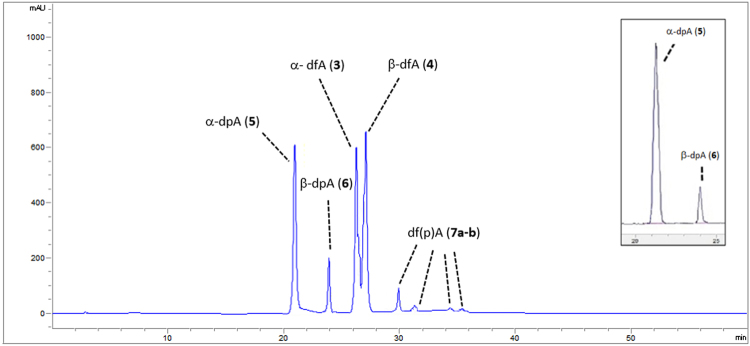
HPLC chromatographic profile for the irradiation of adenine (**1**) and 2-deoxyribose (**2**) in NH_2_CHO and NWA 1465. Peak A (21.026 min): α-dpA (**5**). Peak B (23.944 min): β-dpA (**6**). Peak C (26.176 min): α-dfA (**3**). Peak D (26.684 min) β-dfA (**4**). Peak E (31.347-34.388 min): df(p)A (**7a–b**). The magnification reports the same reaction mixture co-injected with a standard sample of α-dpA (**5**).

The formation of mono- and poly-glycosylated nucleosides was observed: irrespective to experimental conditions, the reaction of adenine (**1**) with 2-deoxyribose (**2**) afforded α-D-2′-deoxy-ribofuranosyl adenine (**3**), β-D-2′-deoxy-ribofuranosyl adenine (**4**), α-D-2′-deoxy-ribopyranosyl adenine (**5**), and β-D-2′-deoxypiranosyl adenine (**6**). Poly-glycosylated N^6^-2′-deoxy-ribofuranosyl-(**7a**) and N^6^-2′-deoxy-ribopyranosyl-2′-deoxyadenosine isomers (**7b**) were also detected and assigned on the basis of comparison with authentic standards prepared as reported^24^ (Fig. 1; Table 1, entries 1–3). Figure 2 shows as an example the UHPLC profile for the irradiation of adenine (**1**) and 2-deoxyribose (**2**) in formamide and NWA 1465.

The complete set of UHPLC-MS/MS data, chromatographic profiles, and selected m/z fragmentation spectra are in SI #4. Chromatographic profiles also reports the magnification of the same reaction mixtures co-injected with standard nucleoside samples. The synthesis and analytical data of standard nucleosides are in SI #5.

**Figure 3:**
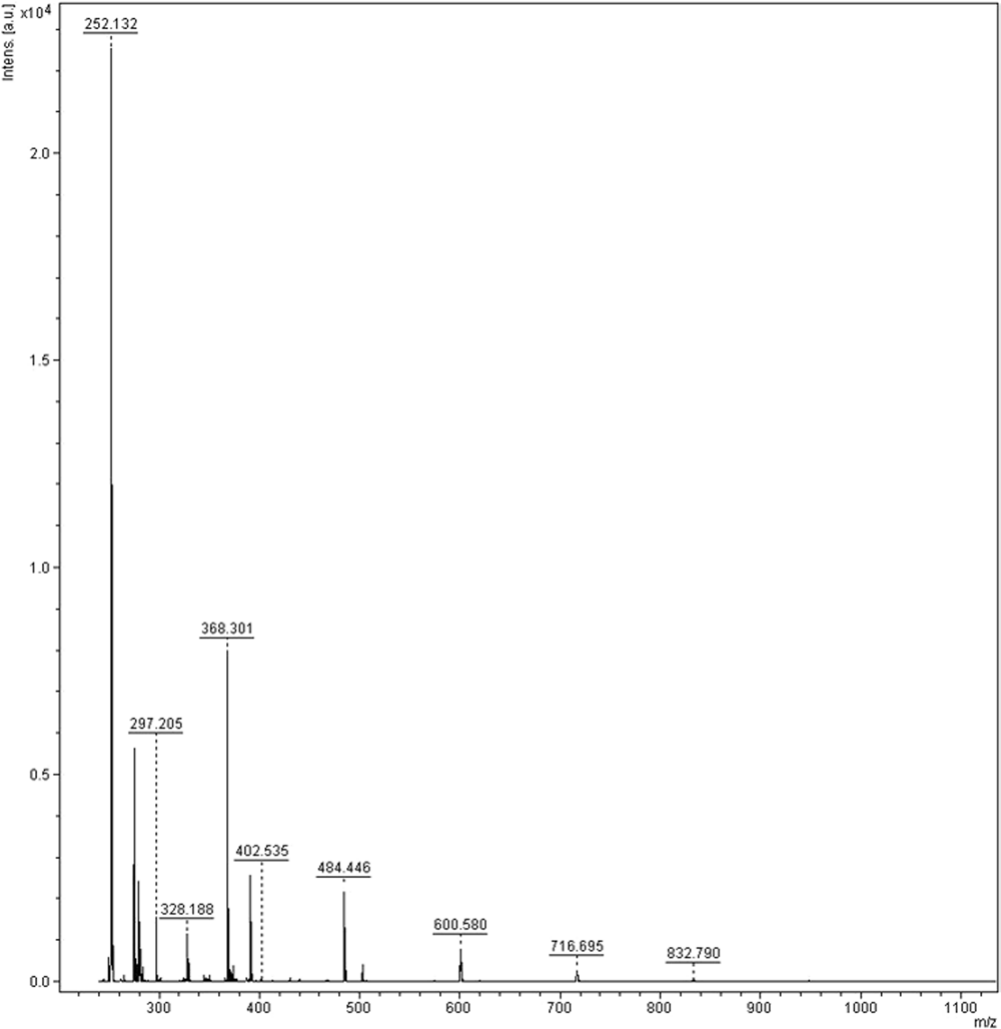
MALDI TOF/TOF analysis of the reaction between adenine (**1**) and 2-D-deoxyribose (**2**) in NH_2_CHO in the presence of NWA 1465. M/z = 252, 2′-deoxy adenosine. M/z = 368 df(p)A (**7a–b**). M/z = 484, 600, 716 and 832 products corresponding to addition of three, four, five and six sugar moieties, respectively.

MALDI TOF analysis (Fig. 3) of the reaction in formamide and NWA 1465 confirmed the presence of products (**3–6**) and (**7a-b**). The complete set of MALDI TOF analyses is SI#6 A-E. Higher molecular weight poly-glycosylated derivatives, corresponding to the addition of three (m/z = 484), four (m/z = 600), five (m/z = 716) and six (m/z = 832) sugar moieties, were also detected. In the irradiation of the solid film, the poly-glycosylation process progressed to a lesser extent, reaching the addition of up to four sugar moieties (SI #6-D). The m/z ions of higher molecular weight poly-glycosylated derivatives were characterized by the loss of a water molecule *per* novel glycosidic bond, as a consequence of the addition/elimination reaction. These data were further confirmed by the MS/MS analysis of the peak at 36.8 min in the HPLC chromatographic profile of the irradiation of adenine (**1**) and 2-deoxyribose (**2**) in formamide, corresponding to a tetra glycosylated derivative (Figure SI #4-I). The absence of the peaks of others poly-glycosylated compounds might be due to their low abundance. In this latter case, the possibility of the presence of non-covalent aggregates, rather than covalently-bound products, cannot be completely ruled out. In extant living systems, poly-glycosylated nucleosides bearing oligosaccharide side chains have been detected in tRNA^Met^ molecules involved in yeasts and plants replication processes^25^. They are products of post-translational modifications in humans and play key roles in cellular physiology and genotoxic stress response^26^.

The reaction of adenine (**1**) with ribose (**9**) afforded α-D-ribofuranosyl adenine **(10**), β-D-ribofuranosyl adenine (**11**), α-D-ribopyranosyl adenine (**12**) and β-D-ribopyranosyl adenine (**13**). In this latter case, the signals of **(10**) and (**13**) overlapped in the UHPLC profile, according to a previously reported chromatographic pattern^19^. Poly-glycosylated derivatives were detected neither by UHPLC-MS/MS nor by MALDI TOF analyses (Fig. 1; Table 2, entries 1–3).

**Table 2:** Reaction of adenine (**1**) with ribose (**9**).

Entry	Conditions	Adenine^a^ (%)	Product yield (%)^c^		
			α-pA (12)	β-fA (11)	α-fA (10) + β-pA (13)
1	Dry state^b^	75	7.3	2.9	14.8
2	FA	63	10.4	5.6	21.2
3	FA/NWA 1465	52	6.1	20.1	16.9

FA = NH_2_CHO. Meteorite = NWA 1465. The reaction provides furanosides (f) and pyranosides (p) as α- and β-isomers. **α-fA = **α-D-ribofuranosyl adenine; **β-fA = **β-D-ribofuranosyl adenine; **α-pA = **α-D-ribopyranosyl adenine; **β-pA** = β-D-ribopiranosyl adenine. ^a^Unreacted adenine. ^b^Obtained after dissolution of ribose in distilled water and successive drying under nitrogen. ^c^The yield was calculated as percentage (%) of nucleoside (mmol) with respect to starting adenine. The data are the mean values of three experiments with standard deviation equal to or less than 0.1%.

2′-Deoxyribosides form more efficiently than ribosides, pyranosides prevail over furanosides, the β-isomer over the α-isomer, and the formation of nucleosides is always enhanced by formamide and by the meteorite. The synthesis of 2′-deoxyribosides and of ribosides progressed from acceptable to high yield. 2-D-deoxyribose (**2**) showed a reactivity higher than D-ribose (**9**). Irrespective of the experimental conditions, the highest yields were obtained in the presence of formamide and NWA 1465. The irradiation of adenine (**1**) and 2-deoxyribose (**2**) in dry state or in formamide afforded pyranosides (**5–6**) in yield higher than that of the furanoside counterparts (**3,4**) (24.9% versus 12.3%, and 42.7% versus 24.4%, respectively. Table 1, entries 1 and 2). Similar results were observed for the reaction of D-ribose, where the pyranoside (**12**) prevailed over furanoside (**11**) [the ratio between (**10**) and (**13**) could not be determined due to overlapping peaks]. These data are in agreement with the prevalence of the pyranoside form for carbohydrates^2^ and^9^ when dissolved in formamide, as evaluated by ^13^C-NMR analysis (Table 3. 83.3% versus 17.7%, and 81.8% versus 18.2%, respectively). Similar results were observed by ^13^C-NMR analysis in water, where the furanose form accounts for 12% of all the ribose in solution^27^ (SI#7D). Pyranosides have been suggested as plausible carbohydrate alternatives in preRNA molecules^28^ instead of ribofuranose units. ^13^C-NMR procedure, spectra and chemical shifts of pyranoside and furanoside isomers in formamide and H_2_O are in SI # 7.

**Table 3:** ^13^C-NMR determination of pyranoside and furanoside isomers of 2-deoxyribose (**2**) and ribose (**9**) in formamide^a^.

Entry	Sugar	Glycoside isomers	Solvent	Amount (%)
1	2-D-deoxyribose (**2**)	β-pyranose	NH_2_CHO (H_2_O)	40.6 (40.0)
2	2-D-deoxyribose (**2**)	α-pyranose	NH_2_CHO (H_2_O)	42.7 (39.0)
3	2-D-deoxyribose (**2**)	β-furanose	NH_2_CHO (H_2_O)	8.0 (9.9)
4	2-D-deoxyribose (**2**)	α-furanose	NH_2_CHO (H_2_O)	9.7 (10.9)
5	D-ribose (**9**)	β-pyranose	NH_2_CHO (H_2_O)	64.9 (62.3)
6	D-ribose (**9**)	α-pyranose	NH_2_CHO (H_2_O)	16.9 (20.5)
7	D-ribose (**9**)	β-furanose	NH_2_CHO (H_2_O)	10.7 (12.1)
8	D-ribose (**9**)	α-furanose	NH_2_CHO (H_2_O)	7.5 (6.7)

^a^Anomeric ratios in formamide and in water calculated on the basis of the intensity of the C1 peaks. Adding to this selectivity, the amount of β-isomers increased in the presence of NWA 1465. β-Isomers usually prevailed in mineral catalysis conditions, presumably due to the preferred attack of the nucleobases from the less hindered side of the carbohydrate adsorbed on the mineral surface^42^.

Noticeably, furanosides **3–4** and **11** were the main isomers in the presence of NWA 1465, showing the important role of the mineral surface at stabilizing the different anomeric forms (Tables 1, 2). The tendency toward stabilizing (**9**) in its furanose form was previously reported on silica, associated with the prevalence of the β over the α stereo-isomer^29^. The formation of silicate five-membered diolate ring chelate^30^, characterized by the appropriate value of the O-C-C-O dihedral angle^31^, was reported to enhance the accumulation of the furanose form^32^. Furthermore, the elimination of water during the drying process increases the amount of the β-anomer^33^. The influence of zinc polydentate complexes on the formation of high proportion of β-furanose (50%) was also reported^29^. The formation of the latter is probably favored for geometrical reasons. From NMR results, the OH group at C5 interacts preferentially with the silica surface, so that the complexation with cations occurs with the OH at C2 and C3. These are more accessible because they are positioned on the opposite side of the molecular plane favoring the stabilization of the β-anomer.

**Figure 4:**
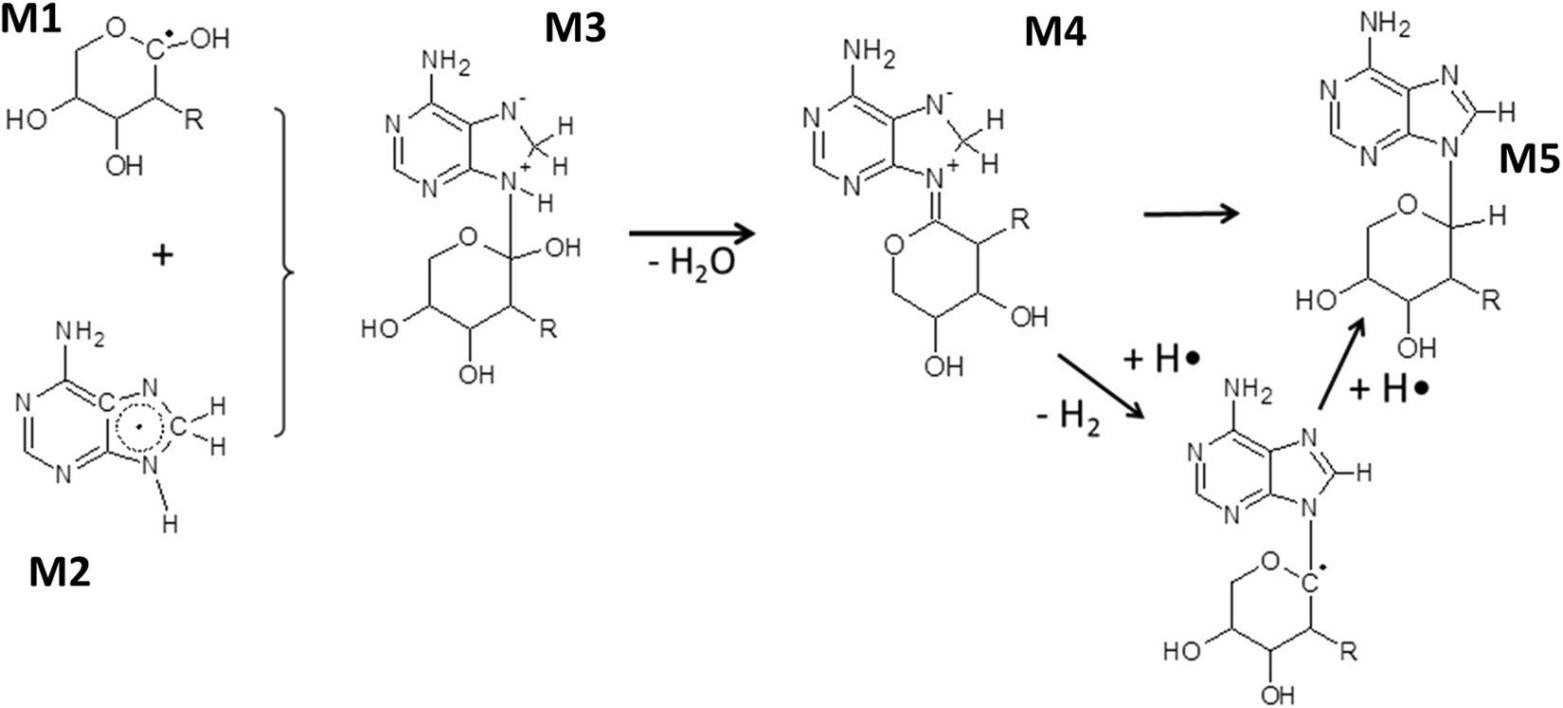
Proposed reaction mechanism for the nucleoside formation assuming adenine base and 2-deoxy ribose in its pyranose forms. R = OH, H for ribose and deoxyribose, respectively. For a more detailed mechanistic model see Fig. SI #8-A.

We next evaluated the possible mechanism of the proton radiation-induced *N*-glycosylation. There is a consensus in the literature that the primary effect of ionizing radiation (like proton irradiation) on sugars is the abstraction of hydrogens from C-H bonds^34–38^. Hydrogen abstraction preferentially occurs on the carbons which are connected to an oxygen because of the electron withdrawing effect by the latter. For this reason, formation of C2-centered radicals of 2-deoxyribose is less likely as compared to that of radicals centered at other carbons^36^. The most stable product in the radiolysis of purine nucleobases is a C8-hydrogenated adduct^39,40^. **M3** formed via the recombination of radiolysis products **M1** and **M2** (Fig. 4 and Figure SI #8-A) loses water in an exothermic reaction step (the computed reaction free energy change for this step is −13.9 kcal/mol). Based on computations using simplified models (for details see SI#8 and SI#10) this proceeds with the very low activation energy of 12.7 kcal/mol if catalytic formamide molecules (in the formamidic acid tautomeric form) are included in the model. The transition state complex for the formamide-catalyzed water abstraction step is depicted in Figure SI#8-B. The product of the dehydration step, compound **M4**, then isomerizes to the nucleotide product in two consecutive reaction steps with participation of two atomic hydrogens. In the first step the C8-position of the nucleobase loses a hydrogen, while in the second step the newly formed radical center at C1′ is saturated with another atomic hydrogen and leads to the nucleoside product **M5**. Since in these two steps the attacking atomic hydrogens form a covalent bond first with the C8 hydrogen and later with the C1′ carbon, both steps are markedly exothermic (the computed reaction free energies changes being −58.3 and −90.9 kcal/mol) and proceed essentially without an activation barrier.

Based on the experimental data, 2-D-deoxyribose (**2**) is more active in the nucleoside formation reaction than D-ribose (**9**). Quantum chemical model calculations (SI #9, Table SI #9-A) suggest that a hydroxyl attached to the adjacent carbon has only a marginal effect on C-H bond dissociation energies. Because of this, there is no reason to assume that significant differences might occur in the rate of C1-radical formation from (**9**) and (**2**). On the other hand, there might be a difference in the degradation rate of these radicals, because of the absence of the 2-OH group in the 2-deoxy form. One of the main degradation channels of polyalcohol radicals is the loss of water from two vicinal hydroxyls located next to the radical center^41^. Because of the lack of the 2-OH, this degradation mechanism is irrelevant to 2-deoxyribose (Figure SI #9-B). Thus, the higher activity of 2-deoxyribose in the glycosidic bond formation might be associated with its enhanced resistance towards degradation.

**Figure 5:**
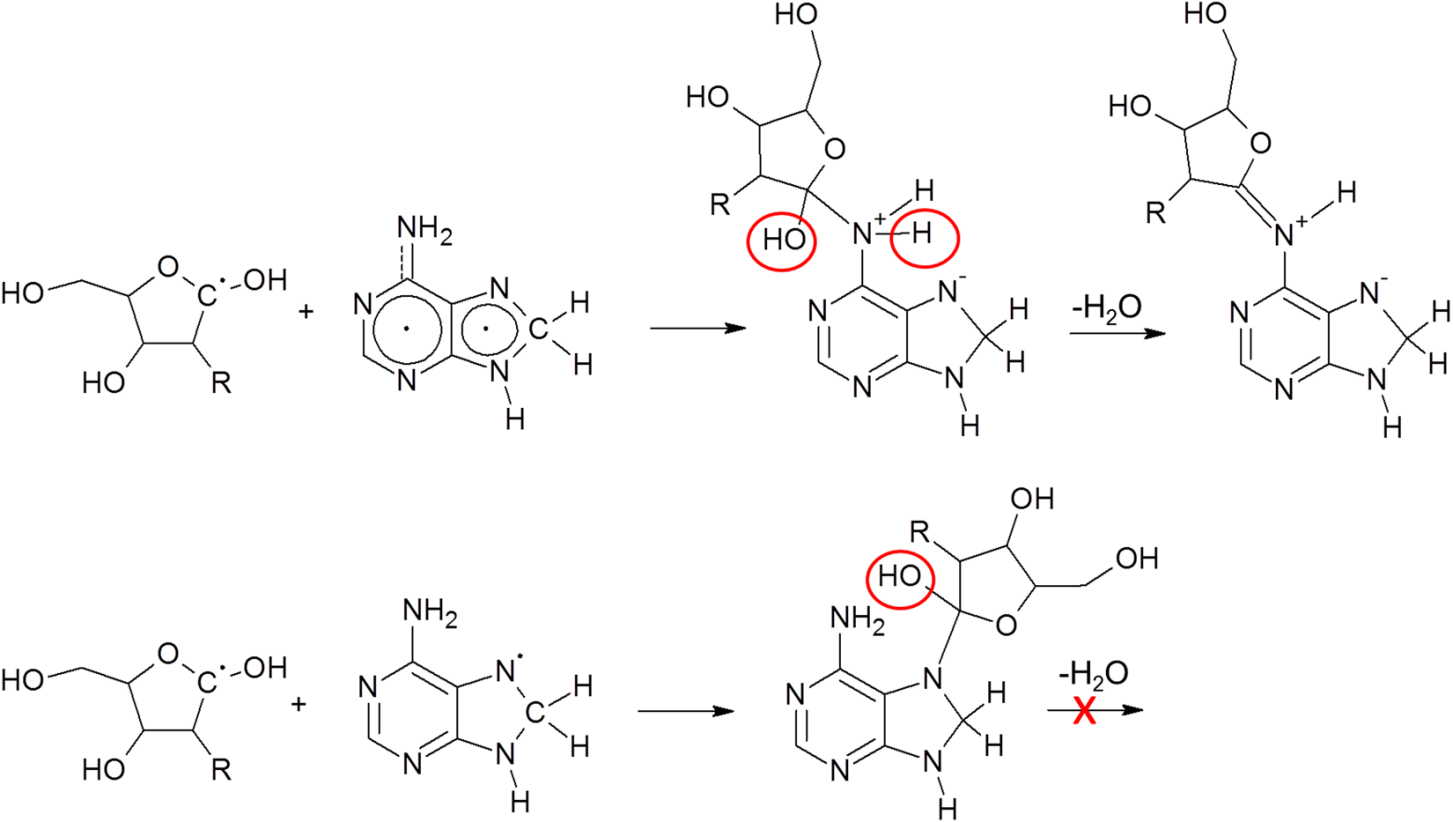
N^6^-glycosidation of nucleobases by a C1-centered ribose radical is made possible by the presence of an H-atom bound to N6. The same mechanism is not possible at N7 because of the lack of covalently bound H.

Finally, we studied the nucleobase regioselectivity of the glycosylation. The glycosylation process under irradiation conditions selectively afforded N9 isomers, which are the isomers that molecular evolution has selected for the formation of nucleic acids. If electron delocalization enables the appearance of the radical character on nitrogens other than the N9 of the aromatic ring (which is the case of adenine), then the water-elimination from the adduct formed between the nucleobase and carbohydrate radicals (similar to the conversion of **M3** to **M4** in Fig. 4) drives the reaction towards the stabilization of the glycosidic bond. This however assumes that one H is available on the nitrogen carrying the radical character (the OH is coming from the carbohydrate radical). If no H is available on the nitrogen (which is for instance the case of N7), then the high-energy adduct cannot be stabilized and will most likely fall apart (Fig. 5). This mechanism is probably responsible for the absence of glycosylation on N1 and N7 of adenine, justifying the observed formation of N^6^-glycosylated adducts.

## Discussion and Conclusions

The major interest of a prebiotically plausible synthesis of nucleosides resides in the hurdles that this topic has met. In its general purport, the term prebiotic refers to compounds or processes that are robust, energetically not excessively demanding, based on commonly available starting materials. Nucleosides can be obtained through de novo synthesis by the oxazoline chemistry^15–17^ or by the formamido pyrimidine chemistry^19^. Both pathways are ingenuous, afford nucleosides in good yields, and provide important data on nucleoside chemistry. The strengths of the oxazoline-based syntheses are their high regio- and stereoselectivity, the weaknesses are the multi-step procedures involved and the fact that they only apply to ribonucleosides. The formamido pyrimidine-based syntheses are high regioselective, moderately stereoselective, multi-step, only apply to purines and afford a mixture of furanosides and pyranosides. The prebiotic worth of these syntheses is inversely proportional to the procedural complexities involved, requiring numerous concentration, purification and supplementation steps, designed to specifically overcome intermediate reactions bottlenecks. How do other approaches, and the results presented here compare with these previous studies?

The difficulty of formation of the β-glycosidic bond between preformed moieties^14^ may be overcome considering alternative routes, as reaction in dry phase or radical chemistry. Considering that this reaction is substantially a dehydration process, Orgel and coworkers^21^ studied it by treating adenine and guanine with D-ribose by heating in dry phase. The reaction yielded 6-ribosylamino adenine and 2-ribosylamino guanine as the only recovered products. This result showed that the reaction in dry phase does not allow for an adequate regioselectivity, since the exocyclic nitrogen atoms are more reactive as nucleophiles than the endocyclic N9- or N1-atoms. Better regioselectivity was observed when performing the reaction in the presence of magnesium salts and inorganic phosphates, affording riboadenosine and riboguanosine in low yield (4% and 9%, respectively) as a mixture of α- and β-isomers.

As for the radical chemistry approach, the data presented in this paper define the pathway of the reaction and the characterization of the products. The reaction is one-pot, high-yield, stereo- and regioselective, and applies both to ribo- and deoxyribonucleosides. In addition, in the presence of one representative part of the meteorite tested, the carbonaceous chondrite NWA 1465, it favors furanosides over pyranosides, which are the anomeric forms present in extant nucleic acids.

## Methods

Formamide, D-ribose, 2-D-deoxyribose and adenine were purchased from Aldrich. NWA 1465 was obtained from Sahara-nayzak, Asnieres sur Seine, France. Gas-chromatography mass-spectrometry (GC-MS) analyses were performed with a LC/GG MS Combo Agilent and Shimadzu GC-MS QP5050A with a Variant CP8944 column (WCOT fused silica, film thickness 0.25 μm, stationary phase VF-5ms, Øί 0.25 mm, lenght 30 m). UHPLC were performed on Ultimate 3000 Rapid Resolution system (DIONEX, Sunnyvale, USA) using Reprosil C18 column (2,5 μm × 150 mm × 2.0 mm). MALDI were performed on Q-Exactive (Thermo). NMR spectra were acquired with a Bruker Avance III 500 spectrometer. Computations were carried out at B3LYP/6-31 + G* level within the COSMO continuum solvent approximation.

### Irradiation experiments

#### General procedure

The solid film (12 mg) of adenine and carbohydrates, or the solution of adenine (0.04 mmol) and carbohydrate (2-deoxyribose or ribose; 0.008 mmol) in formamide (2.0 mL), with or without NWA 1465 meteorite powder (1.0% in weight with respect to formamide, corresponding to mg) were irradiated at 243 K with 170 MeV protons generated by the Phasotron facility of the Joint International Nuclear Institute (JINR; Dubna, Russia) for 3 min. The uniform proton field was bounded to 10×10 cm^2^ by the collimator system. The averaged linear energy transfer (LET) was about 0.57 keV/μm and the calculated absorbed dose was 6 Gy.

Further details of the experiments and computations are given in the Supplementary Material.

## Electronic supplementary material

Supplementary Material
