# Author Correction: Proton irradiation: a key to the challenge of N-glycosidic bond formation in a prebiotic context

**DOI:** 10.1038/s41598-019-40290-6

**Published:** 2019-03-12

**Authors:** Raffaele Saladino, Bruno M. Bizzarri, Lorenzo Botta, Jiří Šponer, Judit E. Šponer, Thomas Georgelin, Maguy Jaber, Baptiste Rigaud, Mikhail Kapralov, Gennady N. Timoshenko, Alexei Rozanov, Eugene Krasavin, Anna Maria Timperio, Ernesto Di Mauro

**Affiliations:** 1 Department of Ecological and Biological Sciences, Via S. Camillo de Lellis, University of Tuscia, 01100 Viterbo, Italy; 2 Institute of Biophysics, Academy of Sciences of the Czech Republic, Královopolská 135, CZ-61265 Brno, Czech Republic; 3 Regional Centre of Advanced Technologies and Materials, Department of Physical Chemistry, Faculty of Science, Palacky University, 17. Listopadu, 771 46 Olomouc, Czech Republic; 4 Sorbonne Universités, UPMC Paris 06, CNRS UMR 7197, Laboratoire de Réactivité de Surface 4 place Jussieu, F-75005 Paris, France; 5 Centre de Biophysique Moleculaire, UPR CNRS4301, Orléans, France; 6 Sorbonne Universités, UPMC Paris06, CNRS UMR 8220, Laboratoire d’Archéologie Moléculaire et Structurale, Paris, France; 7 CNRS Institut des Matériaux de Paris Centre (FR2482), Paris, France; 8 Joint Institute for Nuclear Research, JINR’s Laboratory of Radiation Biology, Dubna, Russia

Correction to: *Scientific Reports* 10.1038/s41598-017-15392-8, published online 07 November 2017

The original version of this Article contained a typographical error in the spelling of the author Mikhail Kapralov, which was incorrectly given as Michail Kapralov. This has now been corrected in the PDF and HTML versions of the Article and in the accompanying Supplementary Information.

