# Supplementary material for: Proton irradiation: a key to the challenge of N-glycosidic bond formation in a prebiotic context
Source: Sci Rep. 2017 Nov 7;7:14709. doi: 10.1038/s41598-017-15392-8 (PMC5677017; doi:10.1038/s41598-017-15392-8)
Supplement: Supplementary file 1 — Supplementary Material [file 41598_2017_15392_MOESM1_ESM.docx]

Supplemetary Material for

**Proton irradiation: a key to the challenge of N-glycosidic bond formation in a prebiotic context**

Raffaele Saladino,^a,*^ Bruno M. Bizzarri,^a^ Lorenzo Botta,^a^ Jiří Šponer,^b,c^ Judit E. Šponer,^b^ Thomas Georgelin,^d,e^ Maguy Jaber,^f^ Baptiste Rigaud,^g^ Mikhail Kapralov,^h^ Gennady N. Timoshenko,^h^ Alexei Rozanov,^h^ Eugene Krasavin,^h^ Anna Maria Timperio,^a^ Ernesto Di Mauro^a,*^

*^a^Department of Ecological and Biological Sciences, Via S. Camillo de Lellis, University of Tuscia, 01100, Viterbo, Italy. ^b^ Institute of Biophysics, Academy of Sciences of the Czech Republic, Královopolská 135, CZ-61265 Brno, Czech Republic.^c^ Regional Centre of Advanced Technologies and Materials, Department of Physical Chemistry, Faculty of Science, Palacky University, 17. Listopadu, 771 46 Olomouc, Czech Republic. ^d^Sorbonne Universités, UPMC Paris 06, CNRS UMR 7197, Laboratoire de Réactivité de Surface 4 place Jussieu, F-75005 Paris-France. ^e^Centre de Biophysique Moleculaire, UPR CNRS4301, Orléans, France. ^f^Sorbonne Universités, UPMC Paris06, CNRS UMR 8220, Laboratoire d’Archéologie Moléculaire et Structurale. ^g^CNRS Institut des Matériaux de Paris Centre (FR2482), Paris, France. ^h^Joint Institute for Nuclear Research, JINR’s Laboratory of Radiation Biology, Dubna, Russia.*

This PDF file includes:

SI # 1. Prebiotic relevance of NH_2_CHO.

SI # 2. Cosmo-origin data of NWA 1465.

SI # 3. Materials and Methods.

SI # 4. LC-MS procedure, HPLC chromatographic profiles, and selected m/z fragmentation spectra.

SI # 5. Analytical data of standards.

SI # 6. Matrix-assisted laser desorption/ionization mass spectrometry MALDI TOF/TOF analysis.

SI # 7. ^13^C-NMR data of 2-D-deoxyribose and D-ribose in NH_2_CHO,

SI # 8. Computational details

Figure-SI # 9. Model illustrating the degradation of the C1-dehydrogenated radical formed from ribose via water loss.

SI # 10. B3LYP/6-31+G* optimized geometries used to evaluate the free energy profile of the *N*-glycosidic bond formation.

References (43-53).

**SI #1: Prebiotic relevance of formamide**

Formamide (NH_2_CHO, the simplest one-carbon amide in nature) is formally the condensation product of HCN and H_2_O, which are two of the most ancient compounds deemed to be among the first molecules formed on our planet (*43*). Formamide is largely diffused in the universe, having been detected in Kparsec-wide interstellar clouds (*6*), and in several space objects (*44*). Space and terrestrial syntheses of formamide under a variety of conditions have been previously described and explained (*45*). For instance, formamide has been produced in the past by reaction of ammonia and formic acid, which was confirmed also by more recent experiments (*46*). While ammonia is generally accepted to be a major component of the primeval atmosphere (*47*), formic acid is the most abundant product formed in the classical Miller–Urey experiment (*48*). At difference from HCN, formamide is liquid between 4 and 210°C, making it particularly suited to temperature-induced concentration phenomena (*49*). Alternatively, it could be concentrated by formation of eutectic phase in ice (*50*). A recent study reports on conditions for its extreme concentration in hydrothermal pores that could enable formation of nucleobases in a prebiotic milieu (*49*). The synthesis of complex organic compounds endowed with biological relevance from formamide under plausible prebiotic conditions has been described and reviewed (*2*). formamide, formed as a consequence of volcanic activity has been suggested to be a friendly chemical environment for the initial steps of prebiotic chemistry, which would resolve the issue of hydrolytic instability of nucleic acid building blocks (*22*).

**SI # 2. Cosmo-origin data of NWA 1465**

NWA 1465 was found in 2001 in the Western Saharan desert. NWA 1465 (*51*) (shock stage, S4) is classified as a type 3 carbonaceous chondrite with flattened chondrules, mineral fragments, and refractory objects in a compact anhydrous matrix of Fe-rich olivine, Ca-rich pyroxene, enstatite, forsterite, troilite, magnetite, FeNi-metal, and weathering products (degree of weathering, W3). NWA 1465 also contains cm-sized Ca, Al-rich inclusions and large inclusions of dark material). The oxygen isotope composition of the bulk of NWA 1465is: δ18O = 4.89‰, δ17O =0.71‰ . The oxygen isotope composition of dark material (δ18O =13.08‰, δ17O = 5.83‰) is not in equilibrium with that of the host meteorite (*52*).

**SI # 3. Materials and Methods**

Preparation of meteorites powder and solid films.

Formamide (Fluka, > 99%) was used without further purification. NWA 1465 was obtained from Sahara-nayzak, Asnieres sur Seine, France. The provider certifies the following composition: the sample is a fragment from a type 3 carbonaceous chondrite with calcium-aluminum inclusions (highly irregularly shaped and composed of spinel, Ti-rich Ca-pyroxene, melilite, and forsterite), forsterite-rich refractory objects, and mineral fragments set into a dark, fine-grained matrix. The mineral fragments are olivines with a mean fayalite content of 5.5 mol % (range Fa 0.4-41.9), pyroxenes with a Fs-content of 2.8 mol % (range Fs 0.8-5.5) and plagioclase (An 83.5, range An 79.6-86.8). The dark, fine-grained matrix is composed of Fe-rich olivine (Fa 43-57.6), Ca-rich pyroxene, enstatite, forsterite, troilite, FeNi-metal, and magnetite. The whole fragment was grinded to powder in order to ensure homogeneity.

Dust (approximately 100 mg) obtained from NWA 1465 was extracted by a two-steps procedure to remove organics. The first consisted in the addition of 1.0 mL 0.1 N NaOH and 3.0 mL of 2:1 chloroform-methanol, the second step in the addition of 1.0 mL 0.1 N sulphuric acid and 3.0 mL of 2:1 chloroform-methanol. Between steps the powder was recovered by centrifugation (6000 rpm, 10 min) and the supernatant phase was decanted. The supernatant contained organics that were soluble in both aqueous and organic solvents at high and low pH ranges, leaving behind the powder of the meteorite. The powder was then pyrolyzed at 600°C to remove the insoluble organic component in the laboratory oven for 1 h.

The solid films were prepared by stirring adenine (0.04 mmol) and the appropriate carbohydrate (2-deoxyribose or ribose; 0.08 mmol) in distilled water (1 ml) for 2 min at 20 °C. The suspension was dried under high-vacuum and inert atmosphere (N_2_) to obtain a white solid film. The films analyzed by HPLC (see below) showed only the presence of adenine. The yield was calculated as percentage (%) of nucleoside (mmol) with respect to converted adenine.

Irradiation experiments

General procedure: the solid film (12 mg) of adenine and carbohydrates, the solution of adenine (0.04 mmol) and carbohydrate (2-deoxyribose or ribose; 0.008 mmol) in formamide (2.0 mL), with or without NWA 1465 meteorite powder (1.0% in weight with respect to formamide, corresponding to mg) were irradiated at 243 Kwith 170 MeV protons generated by the Phasotron facility of the Joint International Nuclear Institute (JINR; Dubna, Russia) for 3 min. The uniform proton field was bounded to 10×10 cm^2^ by the collimator system. The averaged linear energy transfer (LET) was about 0.57 keV/μm and the calculated absorbed dose was 6 Gy. At the end, the work-up of the reaction was performed with a different procedure depending on the nature of the sample: a) solid-films and reaction in formamide alone were directly analysed by HPLC; b) NWA 1465 meteorite powder was removed by centrifugation (6000 rpm, 10 min, HaereusBiofuge).

**SI #4: LC-MS/MS procedure, UHPLC chromatographic profiles, and selected m/z fragmentation spectra**

An Ultimate 3000 Rapid Resolution UHPLC system (DIONEX, Sunnyvale, USA) and a reprosil C18 column REPROSIL-PUR BASIC C18 (2,5 um x 150 mm x2mm) ID were used to perform samples separation eluted with a phase A to phase B gradient lasting 60 minutes. Chromatographic separations were achieved at column temperature of 30 °C; and flow rate of 0.2 ml/min. For positive ion mode (+) MS analyses, a 0-18% linear gradient of solvent A (H_2_O, 0,1% formic acid) to solvent B (acetonitrile) was employed over 50 min returning to 100% A in 10 min. Products were detected by their absorbance at 250 nm. The UHPLC system was coupled with a mass spectrometer Q-Exactive (Thermo). The instrument was used in positive ionization mode with full scan (FS) and a subsequent data dependent acquisition (DDA) mode. The settings for Full scan were as follows: resolution, 70,000; automatic gain control (AGC) target, 3e6; maximum injection time (IT), 100 ms; and scan range, m/z 70–1050. The remaining settings for DDA mode were as follows: resolution, 17,500; AGC target, 1e5; maximum IT, 50 ms; isolation window, m/z 1.0, HCD with stepped normalized collision energy (NCE). The structure of products was further confirmed by co-injection of the reaction mixture with standard samples under similar experimental conditions.

Abbreviations for the products: α-dfA = α-D-2′-deoxy-ribofuranosyl adenine; β-dfA = β-D-2′-deoxy-ribofuranosyl adenine; α-dpA = α-D-2′-deoxy-ribopyranosyl adenine; β-dpA = β-D-2′-deoxy-ribopiranosyl adenine; df(p)A = N^6^ glycosyl-2′-deoxyadenosine isomers; poly-df(p)A=N^6,6^-bis-glycosylated-2′-deoxyadenosine isomers; α-fA = α-D-ribofuranosyl adenine; β-fA = β-D-ribofuranosyl adenine; α-pA = α-D-ribopyranosyl adenine; β-pA = β-D-ribopiranosyl adenine.


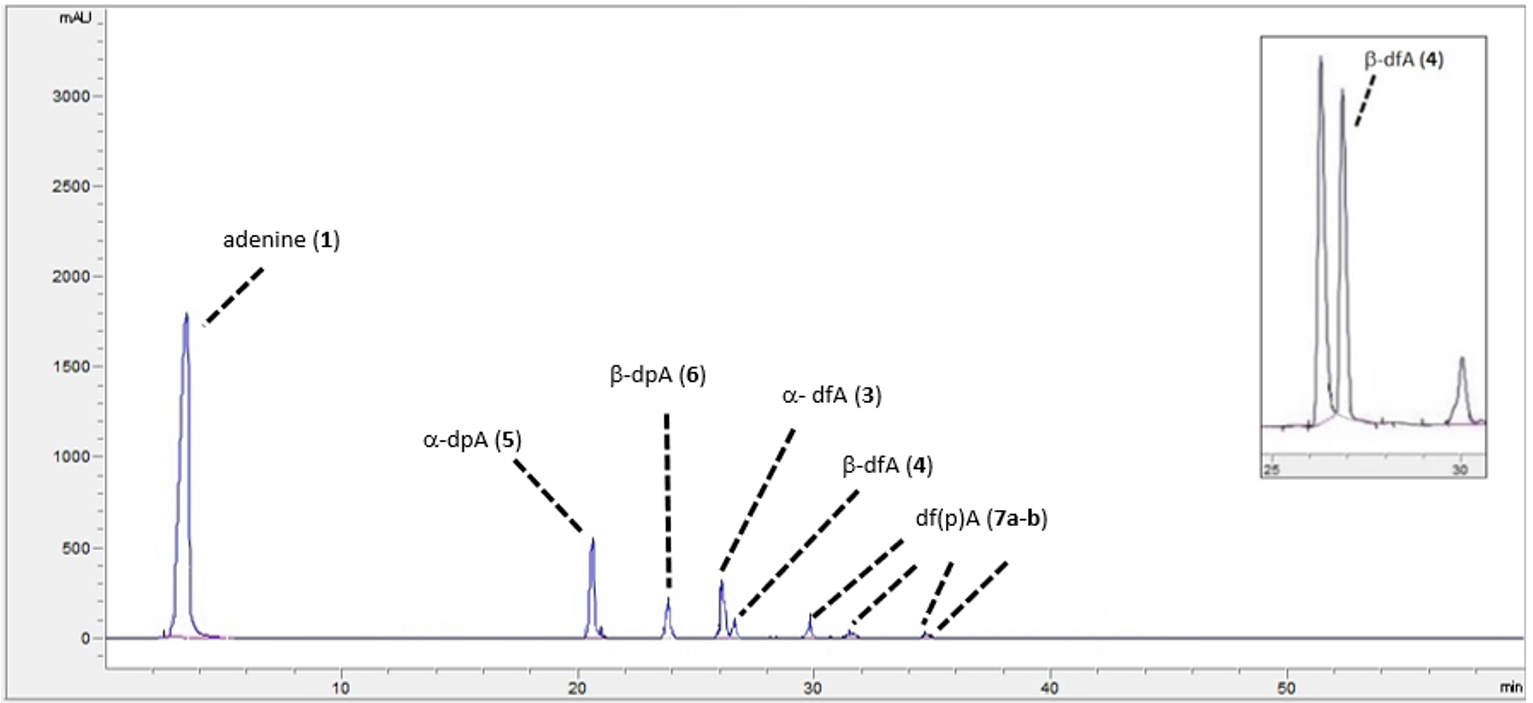


Figure SI #4-A: HPLC chromatographic profile for the irradiation of adenine (**1**) and 2-deoxyribose (**2**) in solid film. Peak A (3.510 min) Adenine (**1**). Peak B (21.208 min): α-dpA(**5**). Peak C (24.045 min): β-dpA(**6**). Peak D (26.287 min): α-dfA(**3**). Peak E (26.867 min) β-dfA(**4**). Peak F (30.021-34.101 min): df(p)A(**7a-b**). The magnification reports the same reaction mixture co-injected with a standard sample of β-dfA(**4**).


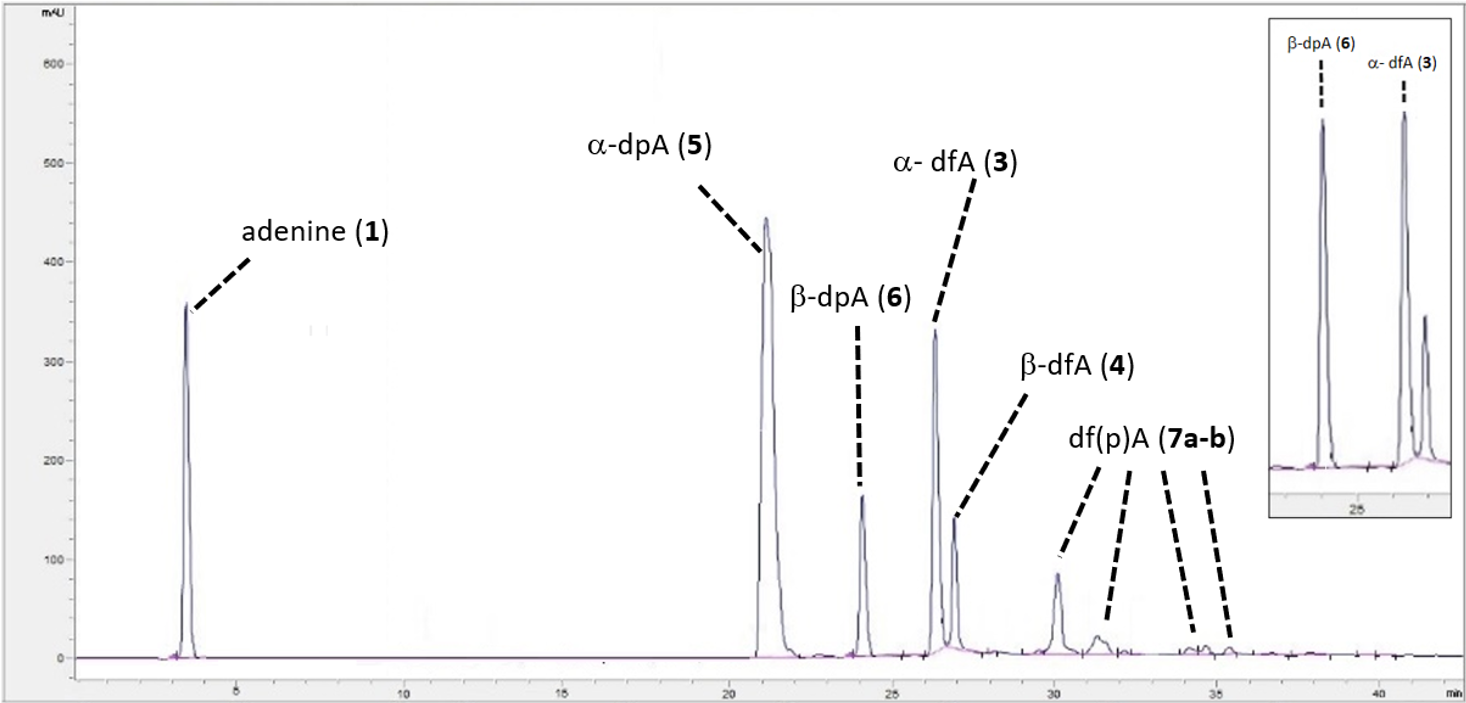


Figure SI #4-B: HPLC chromatographic profile for the irradiation of adenine (**1**) and 2-deoxyribose (**2**) in formamide. Peak A (3.510 min) Adenine (**1**). Peak B (21.208 min): α-dpA(**5**). Peak C (24.045 min): β-dpA(**6**). Peak D (26.287 min): α-dfA(**3**). Peak E (26.867 min) β-dfA(**4**). Peak F (30.021-34.101 min): df(p)A(**7a-b**). The magnification reports the same reaction mixture co-injected with a standard sample of β-dpA(**6**).


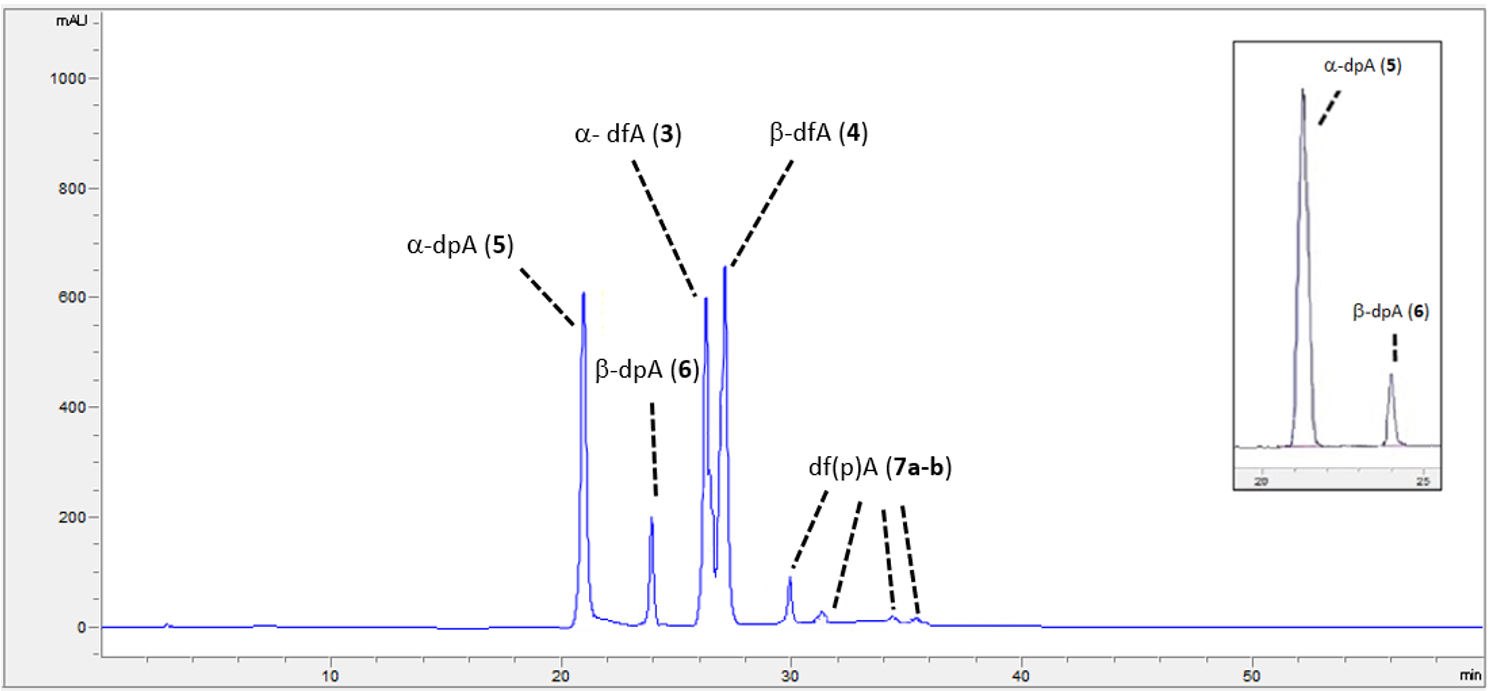


Figure SI #4-C: HPLC chromatographic profile for the irradiation of adenine (**1**) and 2-deoxyribose (**2**) in formamide and NWA 1465. Peak A (21.026 min): α-dpA(**5**). Peak B (23.944 min): β-dpA(**6**). Peak C (26.176 min): α-dfA(**3**). Peak D (26.684 min) β-dfA(**4**). Peak E (31.347-34.388 min): df(p)A(**7a-b**). The magnification reports the same reaction mixture co-injected with a standard sample of α-dpA(**5**).


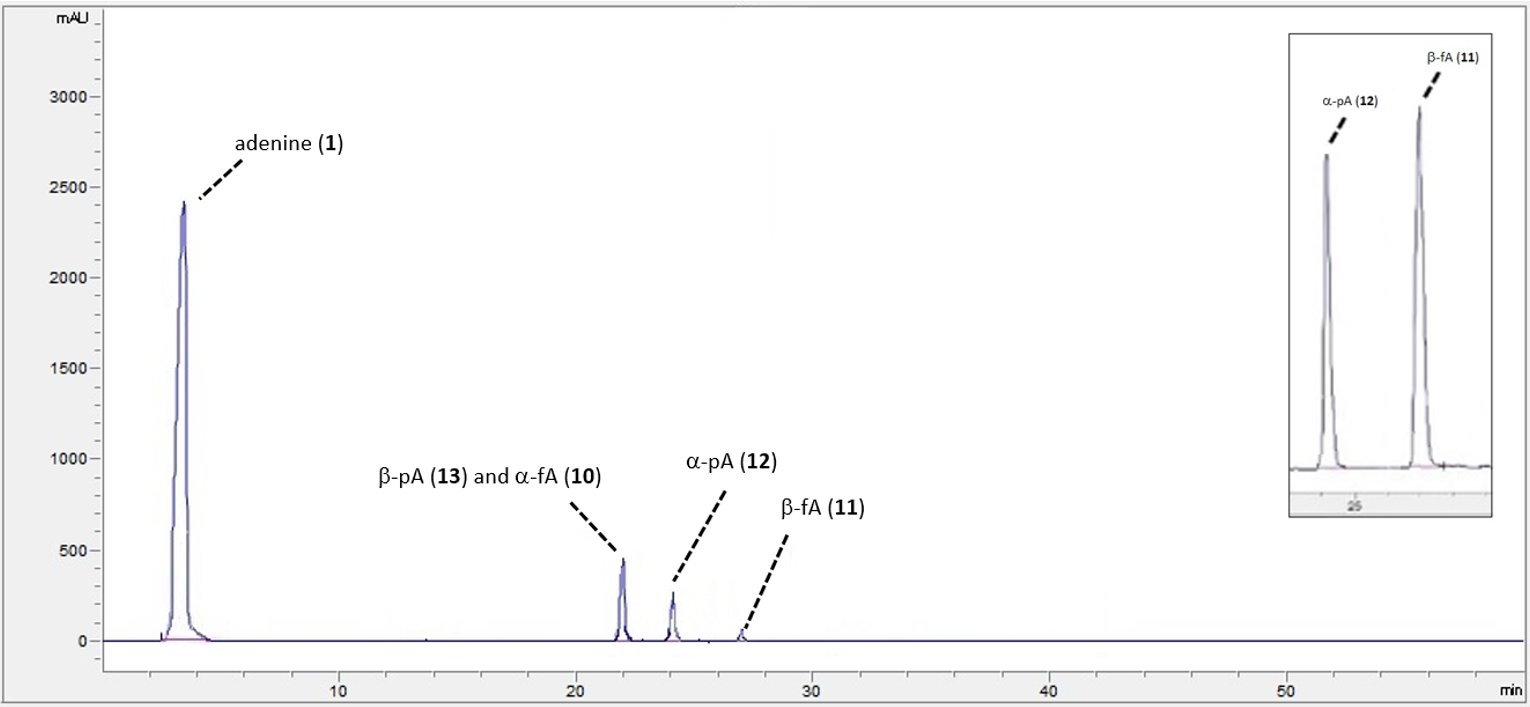


Figure SI #4-D: HPLC chromatographic profile for the irradiation of adenine (**1**) and ribose (**9**) in solid film. Peak A adenine (**1**) overlapped with B (22.008 min): β-pA(**13**) and α-fA(**10**). Peak C (24.165 min): α-pA(**12**). Peak D (27.255 min): β-fA(**11**). The magnification reports the same reaction mixture co-injected with a standard sample of β-fA(**11**).


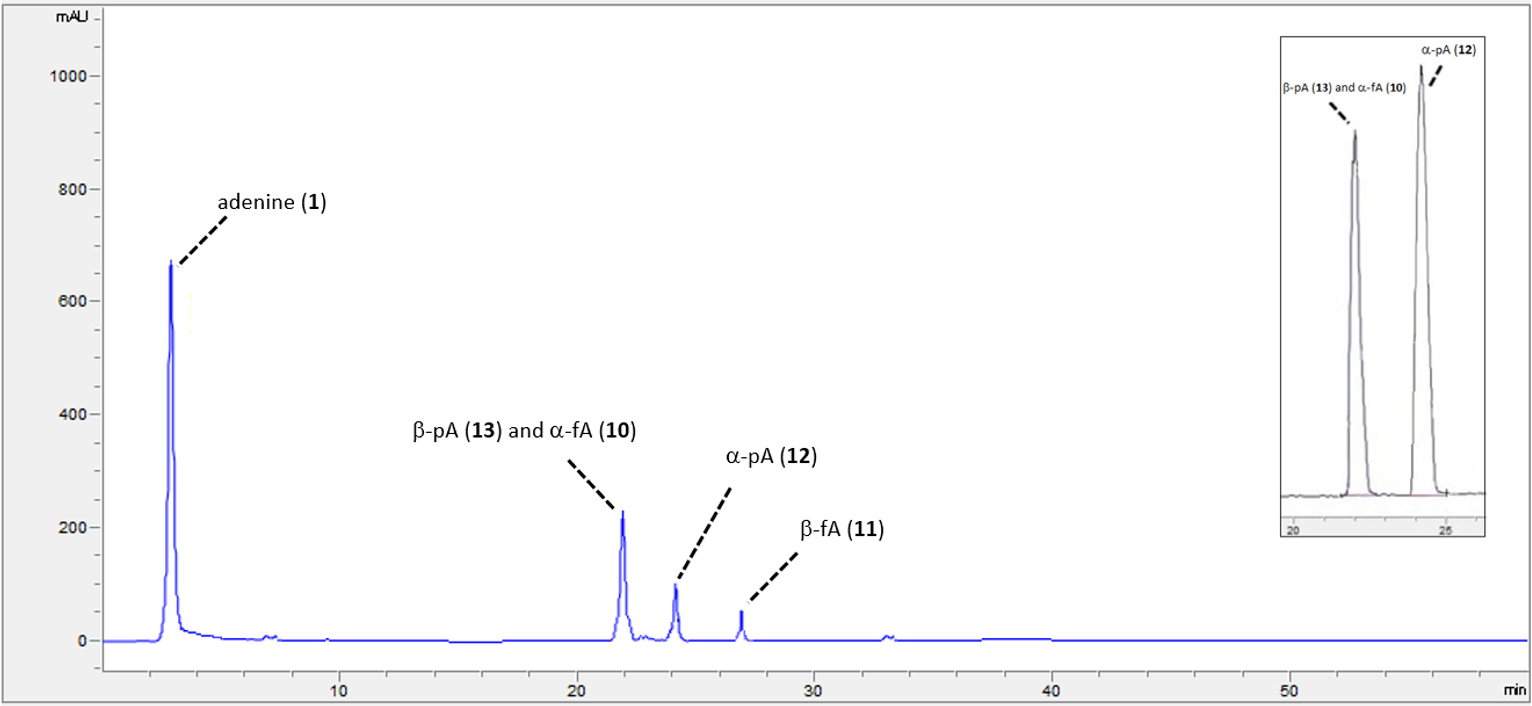


Figure SI #4-E: HPLC chromatographic profile for the irradiation of adenine (**1**) and ribose (**9**) in formamide. Peak A adenine (**1**) (3.510 min). Peak B overlapped with C (22.008 min): β-pA(**13**) and α-fA(**10**). Peak D (24.165 min): α-pA(**12**). Peak E (27.255 min): β-fA(**11**). The magnification reports the same reaction mixture co-injected with a standard sample of α-pA(**12**).


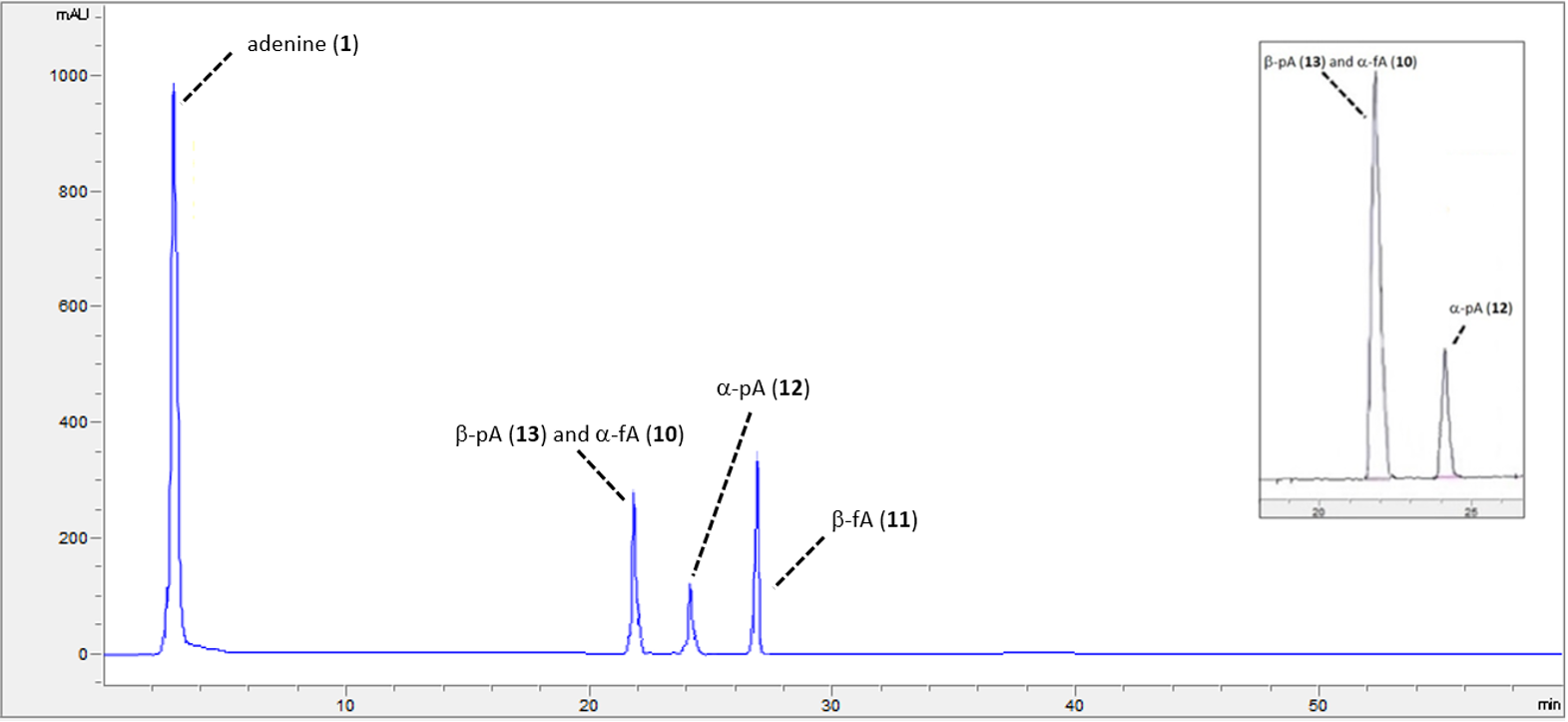


Figure SI #4-F: HPLC chromatographic profile for the irradiation of adenine (**1**) and ribose (**9**) in formamide and NWA 1465. Peak A adenine (**1**) (3.510 min). Peak B overlapped with C (22.008 min): β-pA(**13**) and α-fA(**10**). Peak D (24.165 min): α-pA(**12**). Peak E (27.255 min): β-fA(**11**). The magnification reports the same reaction mixture co-injected with a standard sample of β-pA(**13**).

Selected m/z fragmentation spectra


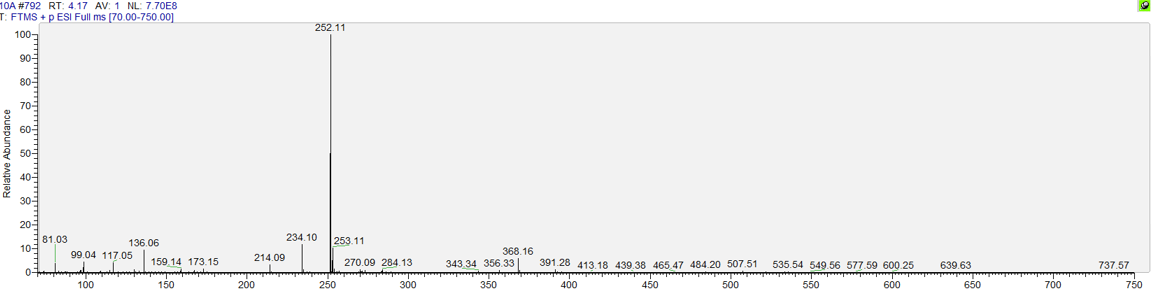


Figure SI #4-G: m/z fragmentation spectrum of α-dfA(**3**) and β-dfA(**4**).


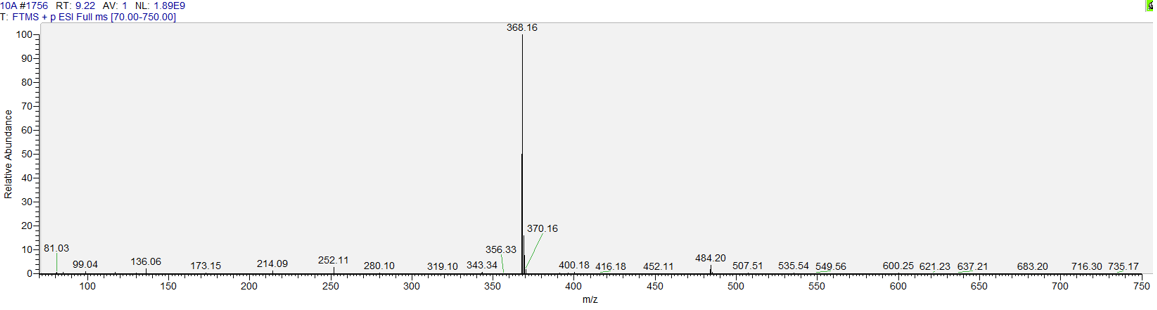


Figure SI #4-H: m/z fragmentation spectrum of df(p)A(**7a-b**).


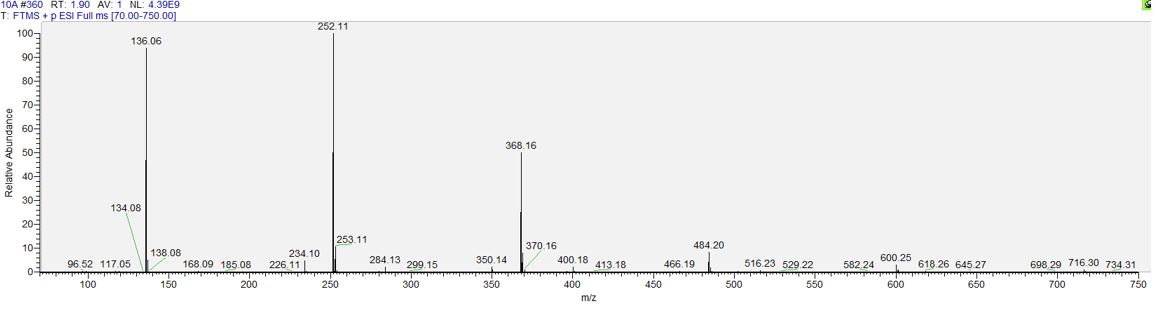


Figure SI #4-I: m/z fragmentation spectrum of the tetra poly-glycosylated derivative corresponding to peak at 36.8 min in the HPLC chromatographic profile of Figure SI #4-B.


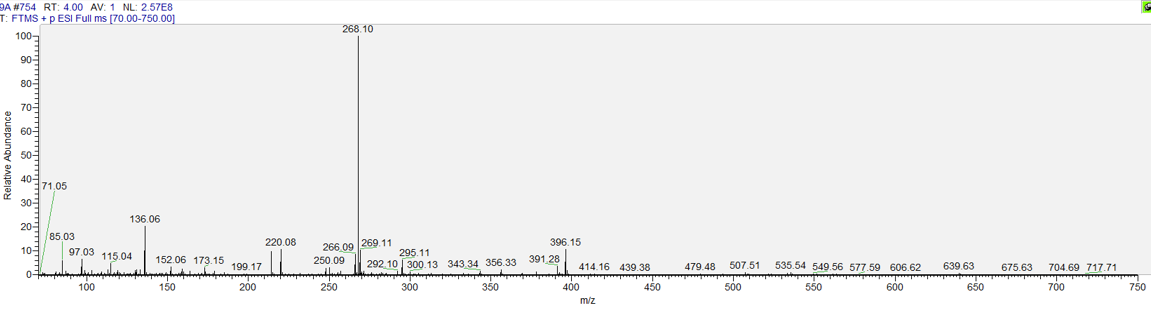


Figure SI #4-L: m/z fragmentation spectrum of β-fA(**11**).

**SI #5: analytical data of standards**

β-D-2′-deoxy-ribofuranosyl adenine, α-D-ribofuranosyl adenine, and** **β-D-ribofuranosyl adenine were purchased from Sigma Aldrich.

**α-D-2**′**-deoxy-ribopyranosyl adenine**

α-D-2′-deoxy-ribopyranosyl adenine was prepared as reported in reference (*24*)

**Spectroscopic data**

**1H-NMR** (400 MHz, DMSO-d_6_) 1.90 (m,1H), 2.50 (m,1H), 3.91-3.65 (m,4H), 4.70 (d 1H), 4.93 (d, 1H), 5.66 (dd, 1H), 7.31(s, 2H), 8.14 (s, 1H), 8.32 (s, 1H) **HRMS** (ESI^+^): calc. for: [C_10_H_13_N_5_O_3_], C,47.84; H, 5.26; N, 27.81; found: C,47.82; H, 5.21; N, 27.86. M.p. 235 °C, [α]_D_ +5.66.

**β-D-2**′**-deoxy-ribopyranosyl adenine**

β-D-2′-deoxy-ribopyranosyl adenine was prepared as reported in reference (*50*)

**Spectroscopic data**

**^1^H-NMR** (400 MHz, DMSO-*d*_6_) 2.01 (m,1H), 2.64 (m,1H), 3.81-3.59 (m,4H), 4.70 (d 1H), 4.96 (d, 1H),5.92 (dd, 1H), 7.25(s, 2H), 8.10 (s, 1H), 8.32 (s, 1H) **HRMS** (ESI^+^): calc. for: [C_10_H_13_N_5_O_3_], C,47.79; H, 5.10; N, 27.72; found: C,47.82; H, 5.11; N, 27.76. [α]_D_ -17.64.

**9-α-D-ribopyranosyl-adenosine** α-pA(**12**)

α-D-ribopyranosyl adenine α-pA(**12**) was prepared as reported in reference (*19*)

**Spectroscopic data**

**^1^H-NMR** (400 MHz, DMSO-*d*_6_) δ = 8.37 (s, 1H; HC8), 8.15 (s, 1H; HC2), 7.31 (s, 2H; NH_2_), 5.76 (s, 1H; HC1′), 5.36 (br, 3H; 3 OH), 3.93 (dd, *J*=12.4, 2.6, 1H; HbC5′), 3.89 – 3.86 (m, 1H; HC2′), 3.84 (t, *J*=3.1, 1H; HC3′), 3.81 – 3.74 (m, 2H; HC4′ and HaC5′). **^13^C-NMR** (100 MHz, DMSO-*d*_6_) δ = 156.00 (C6), 152.57 (C2), 148.86 (C4), 139.79 (C8), 117.84 (C5), 81.14 (C1′), 71.48 (C2′), 69.55 (C5′), 68.53 (C4′), 67.52 (C3′). **HRMS** (ESI^+^): calc. for: [C10H14N5O4]^+^ 268.1040, found: 268.1039 [M+H]^+^.

**9-β-D-ribopyranosyladenine** β-pA(**13**)

β-D-ribopyranosyl adenine β-pA(**13**) was prepared as reported in reference (*19*)

**Spectroscopic data**

**mp**: 241 °C (decomp.); ^1^H**-NMR** (400 MHz, Methanol-*d*4): *δ*= 8.24 (s, 1H; C8), 8.18 (s,1H; C2), 5.75 (d, *J*=9.3, 1H; HC1′), 4.27 (dd, *J*=9.3, 2.8, 1H; HC2′), 4.21 – 4.20 (m, 1H;HC3′), 3.94 (ddd, *J*=10.6, 4.9, 2.6, 1H; HC4′), 3.89 (dd, *J*=10.7, 10.4, 1H; HaC5′), and3.77 ppm (dd, *J*=5.2, 10.4, 1H; HbC5′); **^13^C-NMR** (100 MHz, Methanol-*d*4): *δ*=155.9 (C4), 152.4 (C2), 149.6 (C6), 140.2 (C8), 118.8 (C5), 80.8 (C1′), 71.4 (C3′), 68.8 (C2′),66.7 (C4′), and 65.2 ppm (C5′); **IR** (cm^-1^): ν ~= 3215 (br, m), 2939 (w), 1663 (s), 1609(m), 1577 (m), 1490 (w), 1417 (m), 1333 (m), 1291 (w), 1255 (m), 1150 (w), 1114 (m),1088 (s), 1047 (vs), 1045 (s), 977 (m), 885 (m), 795 (w), 729 (m); **HRMS** (ESI^+^): calc. for: [C10H14N5O4]^+^ 268.1040, found: 268.1039 [M+H]^+^.

***N*^6^-(2**′**-deoxy-ribofuranosyl)-2**′**-deoxyadenosine and *N*^6^-(2**′**-deoxy-ribopyranosyl)-2**′**-deoxyadenosine** (**7a-b**).

*N*^6^-(2′-deoxy-ribofuranosyl)-2′-deoxyadenosine and *N*^6^-(2′-deoxy-ribopyranosyl)-2′-deoxyadenosine df(p)A(**7a-b**) were prepared in the form of inseparable mixture as reported in reference (*24*). Briefly, 2′-deoxyadenosine monohydrate (154 mg, 0.57 mmol) and 2-deoxy-D-ribose (351 mg, 2.62 mmol) were dissolved in glacial acetic acid (300 μl) and methanol (700μl). The mixture was incubated at 37^◦^C for 72 h, the solvent was removed under reduced pressure and the crude was subjected to column silica gel eluted with a gradient of 5–12% methanol in dichloromethane to afford df(p)A(**7a-b**) (115 mg, 54% yield). As expected, the NMR spectra of df(p)A(**7a-b**) showed all signals referred to the equilibrium between the pyranoside and furanoside forms.

df(p)A(**7a**)df(p)A(**7b**)

**^1^H NMR** (400 MHz, D_2_O) 8.29 (0.63H, s, H8), 8.29 (0.29H, s, H8), 8.28 (0.08H, s, H8), 8.26 (0.29H, s, H2), 8.25 (0.55H, s, H2), 8.25 (0.08H, s, H2), 8.24 (0.08H,s, H2), 6.43 (1H, t, *J*=6.9Hz, dR1), 6.28 (0.08H, br s, H1),6.21 (0.08H, br s, H1), 5.81 (0.29H, brs, H1), 5.48 (0.55H, brs, H1), 4.64 (1H, m, H3), 4.47 (0.08H, m, H3), 4.44(0.08H, m, H3), 4.29 (0.29H, m, H3), 4.17 (1H, m, H4), 4.10 (0.08H, m, H4), 4.07 (0.55H, m, H3), 4.02 (0.08H,m, H4), 3.94 (0.55H, m, H5), 3.90 (0.84H, m, H4), 3.84(0.29H, m, H5), 3.83 (1H, m, H5), 3.79 (0.55H, m, H5), 3.77 (1H, m, H5), 3.76 (0.29H, m, H5), 3.70 (0.08H, m, H5), 3.69 (0.08H, m, H5), 3.65 (0.08H, m, H5), 3.64 (0.08H, m, H5), 2.80 (1H, m, H2), 2.66 (0.08H, m, H2), 2.55 (1H, m, H2), 2.39 (0.08H, m, H2), 2.33 (0.08H, m, H2), 2.24 (0.29H, m, H2), 2.16 (0.08H, m, H2), 2.13 (0.55H, m, H2), 2.08 (0.29H, m, H2), 2.07 (0.55H, m, H2); **^13^C NMR** (100 MHz, D_2_O) 156.1 (C6), 156.0 (C6), 155.9 (C6), 154.9 (C2), 154.8 (C2), 154.8 (C2), 154.7 (C2),151.3 (C4), 143.5 (C8), 143.3 (C8), 122.2 (C5), 122.1(C5), 122.0(C5), 90.1 (C4), 90.1 (C4), 88.6 (C4), 87.9 (C4),87.4 (C1), 87.3 (C1), 88.6 (C1), 80.3 (C1), 77.9(C1), 74.3(C3), 73.9 (C3), 73.8 (C3), 70.3 (C3), 69.6 (C5),69.3 (C4), 69.2 (C4), 68.5 (C3), 66.1 (C5), 64.7(C5),64.4 (C5), 63.9 (C5), 41.7 (C2), 41.7 (C2), 41.6 (C2), 37.1 (C2), 35.6 (C2).


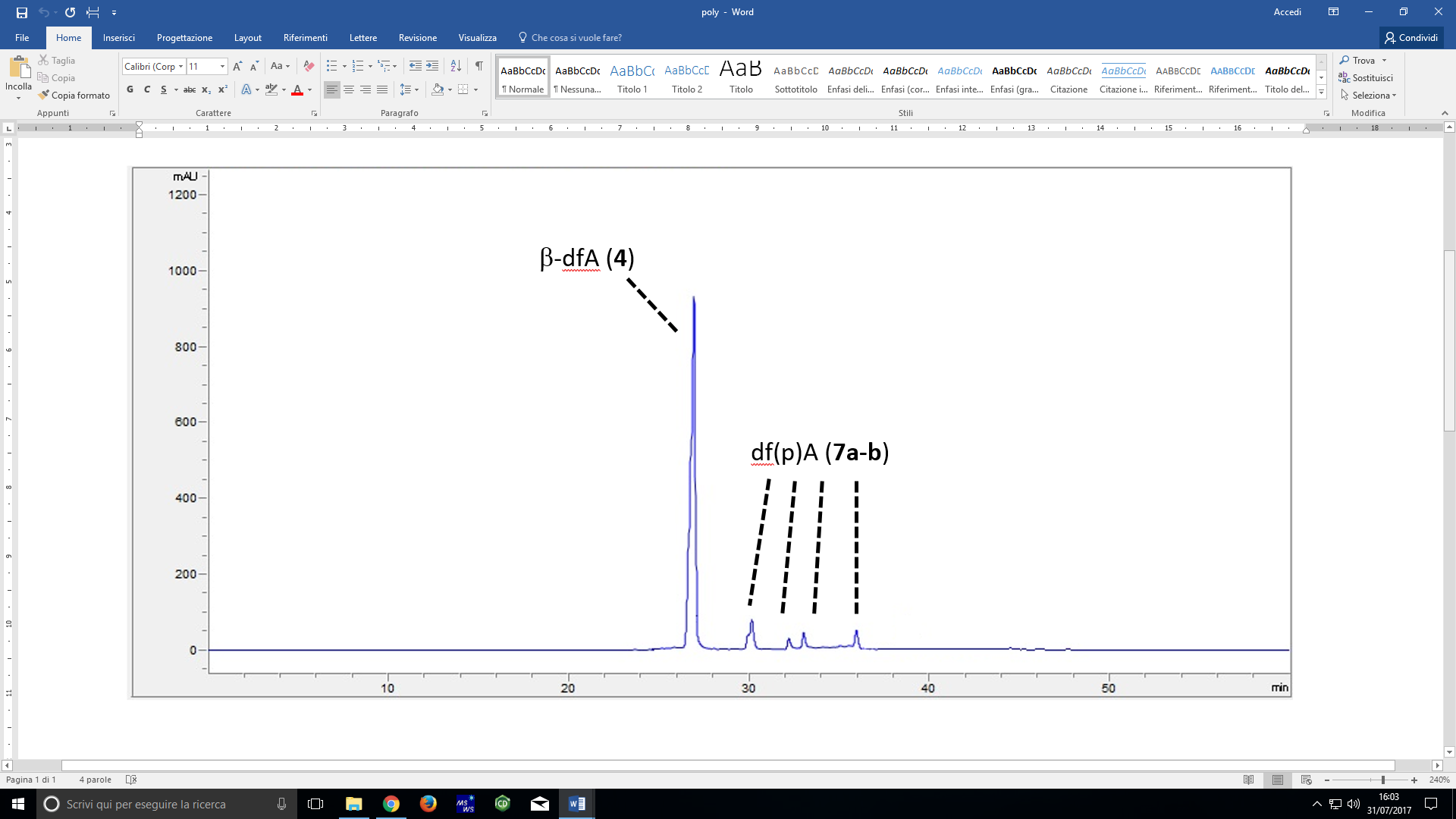


Figure SI #5-A: UHPLC chromatogram of df(p)A(**7a-b**). In accordance with data reported in the literature (*18*), the UHPLC profile of df(p)A(**7a-b**) shows four defined signals referred to peaks 2 and 4 as major isomers, and peaks 3 and 5 as minor isomers. Peak 1 is 2′-deoxyadenosine produced by partial degradation of df(p)A(**7a-b**).

**SI # 6: Matrix-assisted laser desorption/ionization mass spectrometry MALDI TOF/TOF analysis**.


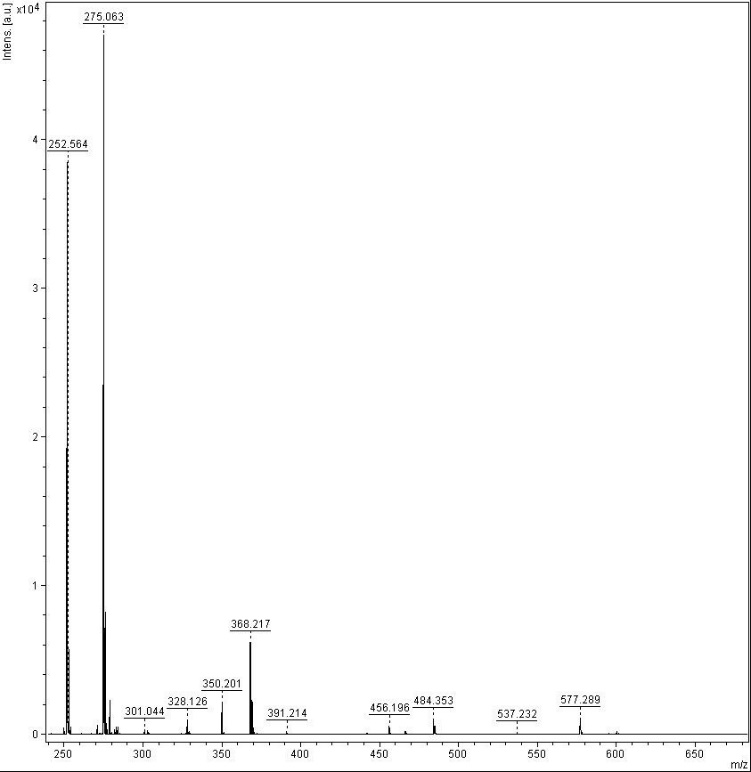
Mass spectra were automatically acquired, using the Autoflex III MALDI-TOF mass spectrometer equipped with a nitrogen laser, working in linear positive mode and controlled by the dedicated custom-made software FlexControl. The samples were mixed with the MALDI matrix (3-hydroxypicolinic acid) and the mixture was applied onto a MTP AnchorChip TM var/384 TF MALDI target. The mass spectra were calibrated externally by using synthetic peptide standards II and alpha-cyano-4-hydroxycinnamic acid (HCCA), used also as the MALDI matrix for calibration runs. Each sample was exposed to a laser with a frequency of 100 Hz and an output of between 30 and 40%. Spectra were analysed using the Flex Analysis software (Bruker Daltonics, Bremen, Germany).

Figure SI #6-A: MALDI TOF/TOF analysis of the reaction between adenine (**1**) and 2-D-deoxyribose (**2**) in solid film. M/z= 252 and 275 correspond to 2′-deoxy adenosine.


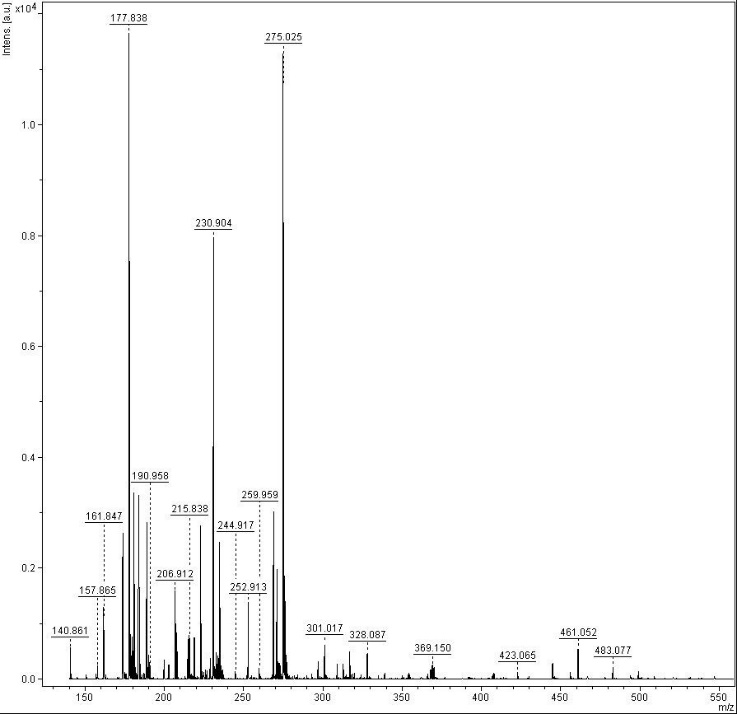


Figure SI #6-B: MALDI TOF/TOF analysis of the reaction between adenine (**1**) and 2-D-deoxyribose (**2**) in NH_2_CHO. M/z= 252 and 275 correspond to 2′-deoxy adenosine.


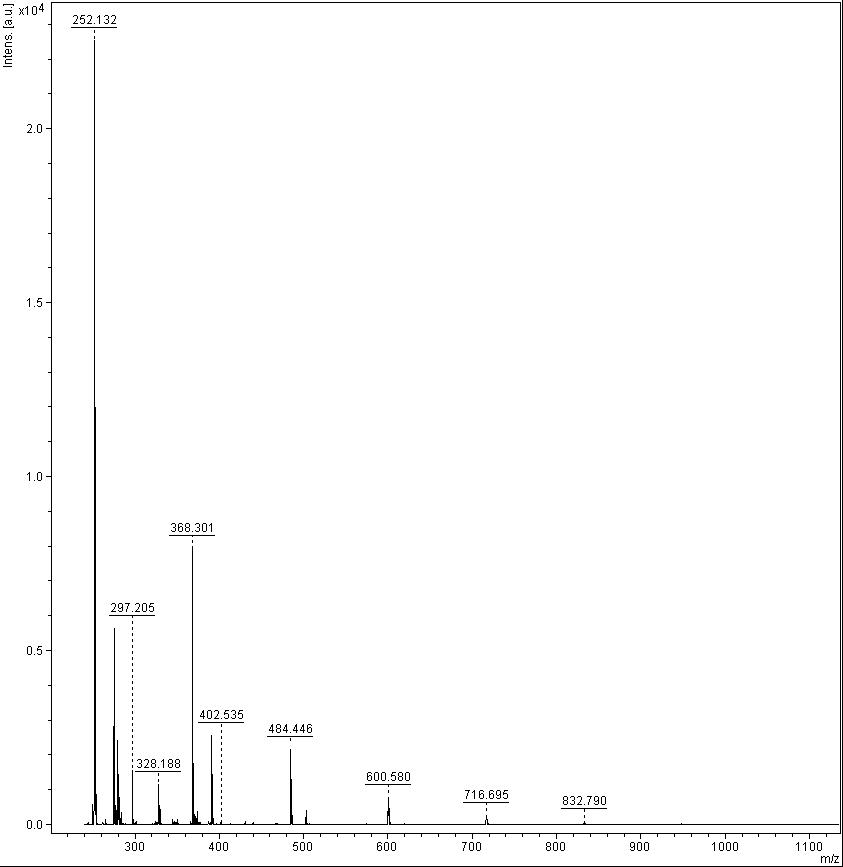


Figure SI #6-C: MALDI TOF/TOF analysis of the reaction between adenine (**1**) and 2-D-deoxyribose (**2**) in NH_2_CHO in the presence of NWA 1465. M/z= 252, 2′-deoxy adenosine. M/z= 368 df(p)A(**7a-b**). M/z= 484, 600, 716 and 832 products corresponding to addition of three, four, five and six sugar moieties, respectively.


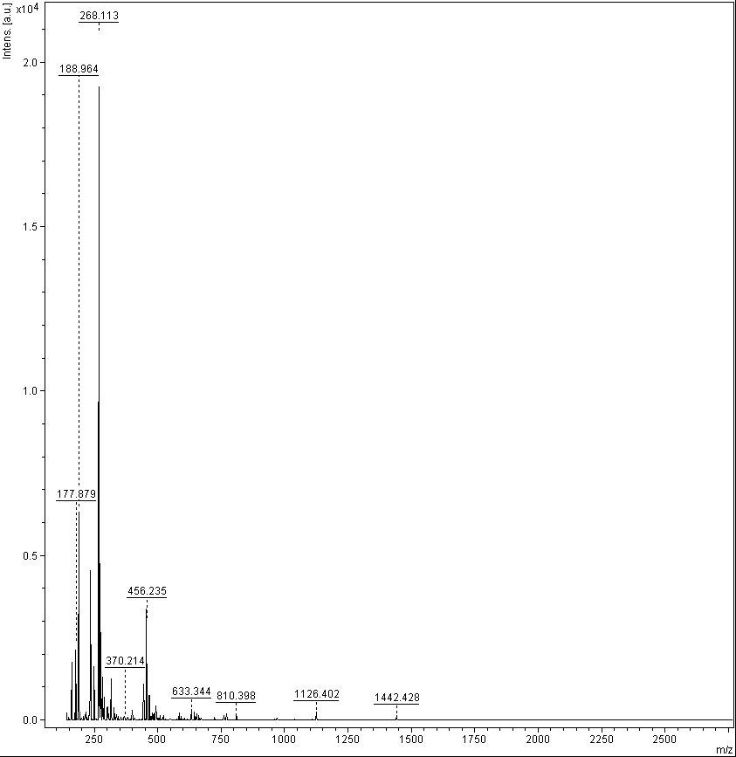


Figure SI #6-D: MALDI TOF/TOF analysis of the reaction between adenine (**1**) and D-ribose (**9**) in solid film M/z= 268 adenosine.


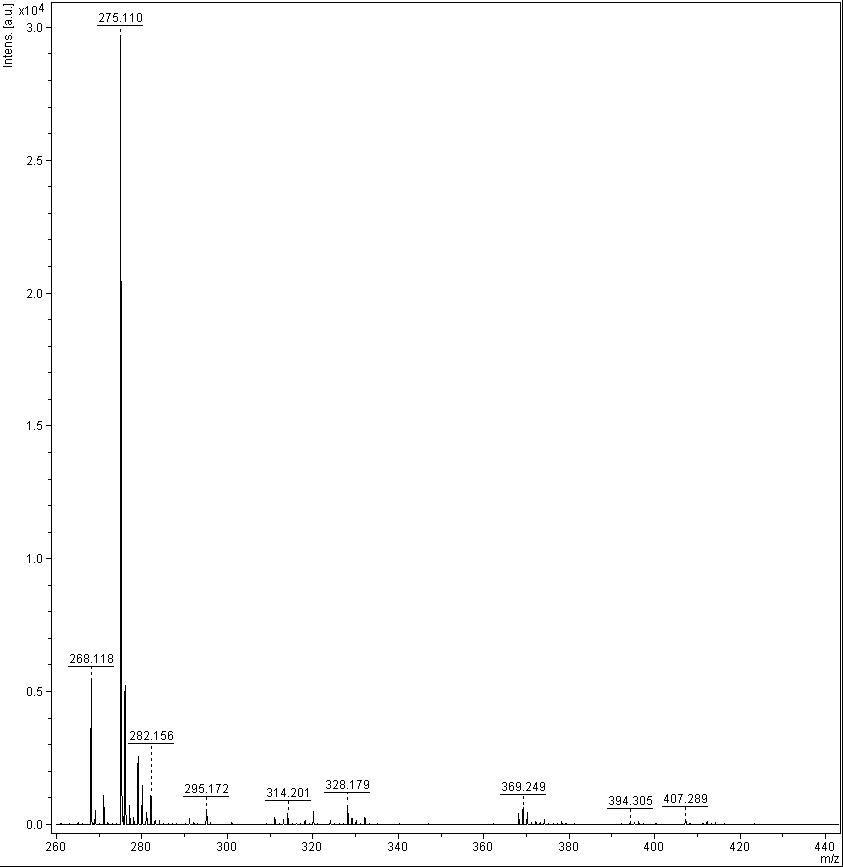


Figure SI #6-E: MALDI TOF/TOF analysis of the reaction between adenine (**1**) and D-ribose (**9**) in NH_2_CHO in the presence of NWA 1465. M/z= 268 adenosine.

**SI # 7. ^13^C-NMR data of 2-D-deoxyribose (2) and D-ribose** (**9**) **in Formamide**.

General procedure. NMR spectra were acquired with a Bruker Avance III 500 spectrometer at a frequency of 500.13 MHz equipped with a 5 mm double resonance broadband probe. Samples were packed in a 5mm medium wall Pyrex Tube with Pressure Valve Sample. Chemical shifts were calibrated using the carbon of TMS (0 ppm) as an external standard. The experiment using 30° pulse with a 1H decoupling waltz16 was recorded in exactly the same condition of temperature and acquisition time. The number of scans to obtain the spectra depended on the S/N obtained for each sample with 2s recycle delay. Spectra were processed with a zero filling factor of 1 and with an exponential decay corresponding to 1 Hz line broadening in the transformed spectra. Only spectra with the same line broadening are directly compared. The T1 of each carbon was measured and the maximum value found is 2.5 s. In our case, we used a recycling time of 2 s to which we must add 1 s of acquisition time, i.e., Tr = D1+aq = 3 s. Thus, according to the equation:

θ=arccos⁡*(e-TrT1)*

we should use an angle of 72 ° to have the total relaxation. In our experiments, we used an angle of Ersnt of 30 °, thus we can say that between each scan we are back to equilibrium.

2-D-deoxyribose, NH_2_CHO

Figure SI #7-A: ^13^C-NMR of 2-D-deoxyribose (**2**) in formamide.

D-ribose, NH_2_CHO

Figure SI #7-B: ^13^C-NMR of D-ribose (**9**) in formamide.

2-D-deoxyribose, H_2_O

Figure SI #7-C: ^13^C-NMR of 2-D-deoxyribose (**9**)in H_2_O.

Figure SI #7-D: ^13^C-NMR of D-ribose (**9**) in H_2_O.

Concerning the Noe effect, the inverse-gated decoupling sequence is more efficient to eliminate the effect but the acquisition time is very long. For this reason we used a power gated decoupling. Our results obtained for the ribose in water were in good accordance with data from the literature (27), confirming the relevance of the sequence that we used in our analysis. In the Ortiz paper (27), in which the inverse-gated decoupling was used, the quantification of each carbon is the following (in parentheses, our results):

β-pyranose 62 % vs 60.9 % (62 %)

α-pyranose 20 % vs 21.1 % (20.5 %)

β-furanose 12 % vs 11.6 % (12 %)

α-furanose 6 % vs 6.3 % (6.5 %)

In order to verify the Ortiz results, we have performed the same experiment, using an inverse-gated decoupling sequence. The results are the following (in parentheses, our results with the power gated decoupling sequence):

β-pyranose 60.9 % (62 %)

α-pyranose 21.1 % (20.5 %)

β-furanose 11.6 % (12 %)

α-furanose 6.3 % (6.5 %)

Figure SI #7-E: ^13^C NMR of ribose in water: inverse-gated decoupling sequence

This additional result confirms that the power gated decoupling sequence that we used in our analysis is correct.

**SI #8: Computational details.**

Computations were carried out at B3LYP/6-31+G** level within the COSMO continuum solvent approximation assuming NH_2_CHO as solvent. All parameters have been relaxed in the course of the geometry optimizations. The free energies (G) of the studied compounds were estimated from the total electronic energy (E^tot^) computed at CCSD(T)/6-31+G* level using the B3LYP/6-31+G** optimized geometries (abbreviated as CCSD(T)/6-31+G**//B3LYP/6-31+G** level) and from the corresponding thermal and entropic correction terms to the Gibbs free energy (δG) from frequency calculations conducted within the harmonic approximation at T=298K: G = E^tot^+ δG. All of the calculations were performed using the Gaussian G09 computer code (*53*).

**
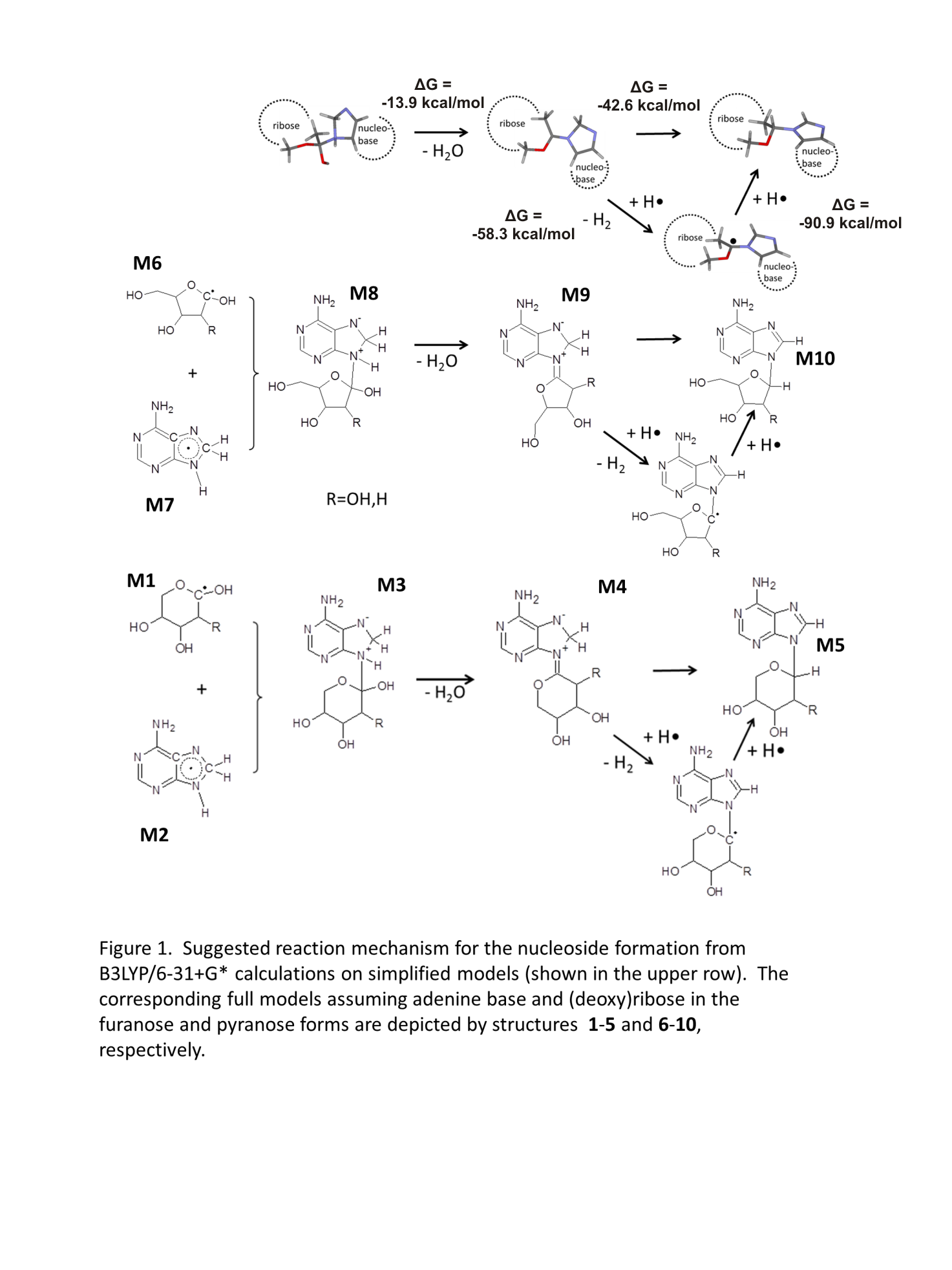
**

**Figure SI #8-A.** Suggested reaction mechanism for the nucleoside formation from B3LYP/6-31+G** calculations on simplified models (shown in the upper row). The corresponding full models assuming adenine base and 2-deoxyribose in the pyranose and furanose forms are depicted by structures M1-M5 and M6-M10, respectively. R=OH, H for ribose and 2-deoxyribose, respectively. ΔG refers to the reaction free energy change of the individual reaction steps and was obtained from CCSD(T)/6-31+G** single point calculations using the B3LYP/6-31+G** optimized geometries.

**
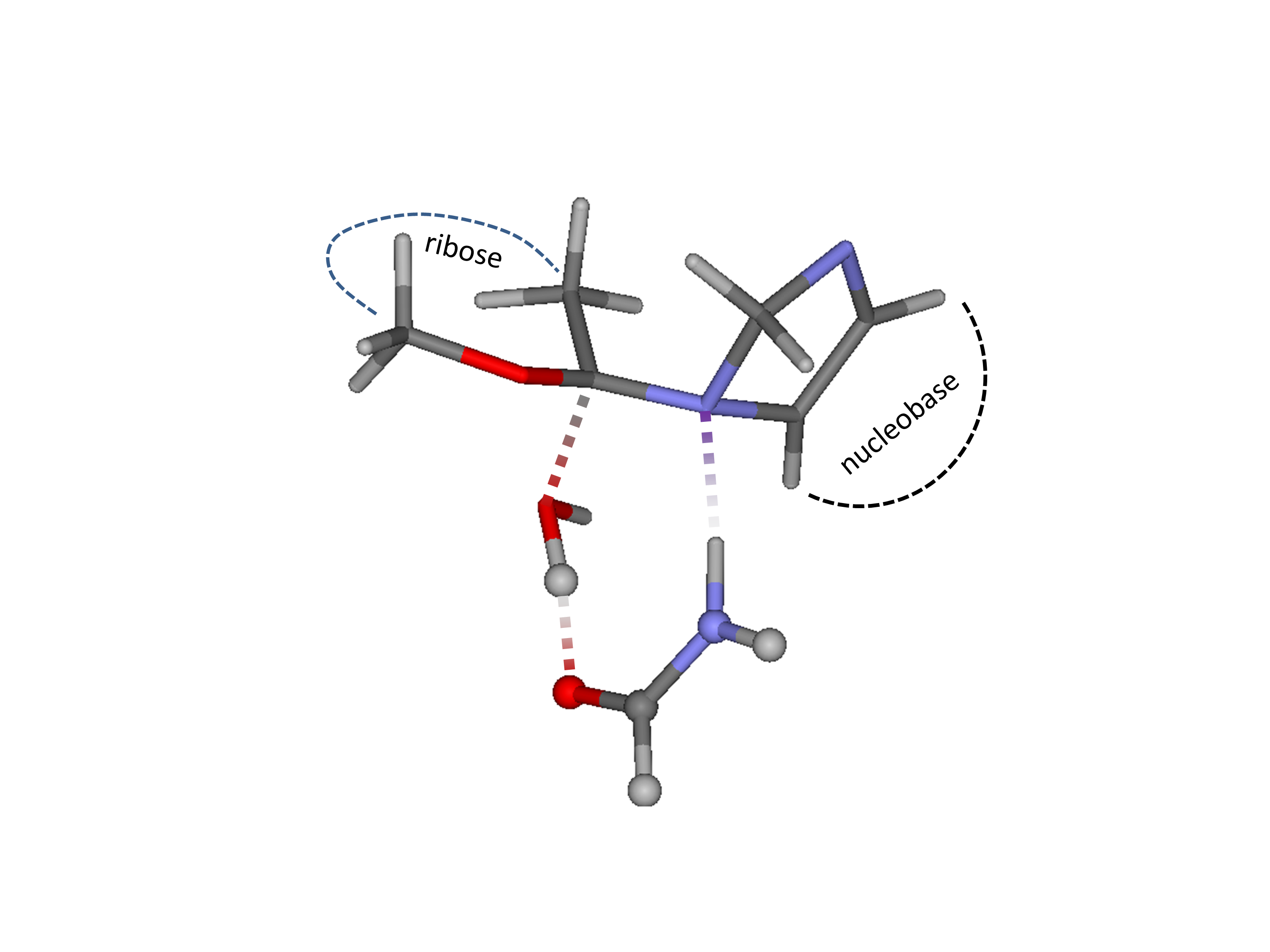

Figure SI #8-B.** B3LYP/6-31+G** optimized geometry of the transition state complex for water abstraction from the simplified model of compounds M3 and M8 (see Figure SI#8-A).

**SI #9. Model calculations of C-H bond dissociation energies.**

**Table SI #9-A**. Reaction free energy changes for C-H bond dissociation reactions (ΔG, kcal/mol) from CCSD(T)/6-31+G**//B3LYP/6-31+G** calculations

| Reaction | ΔG |
| --- | --- |
| CH_3_-CH_3_ = CH_3_-C^•^H_2_ + H^•^ | 94.9 |
| CH_3_-CH_2_OH = CH_3_-C^•^H-OH + H^•^ | 90.3 |
| CH_2_OH-CH_2_OH = CH_2_OH-C^•^H-OH + H^•^ | 90.9 |

As the second and third rows of Table SI #9-A show bond dissociation energies of C-H bonds in a -CH_2_OH moiety are the same within the error margin of the calculations (<1 kcal/mol) independently whether or not a hydroxyl is connected to the carbon *next* to the site of H-abstraction. In contrast, as the first line of the table shows, lack of electron withdrawing groups, such as OH, directly connected to the site of H-abstraction makes the C-H bond dissociation energy markedly higher. This is in line with the conclusions of another earlier study (37).


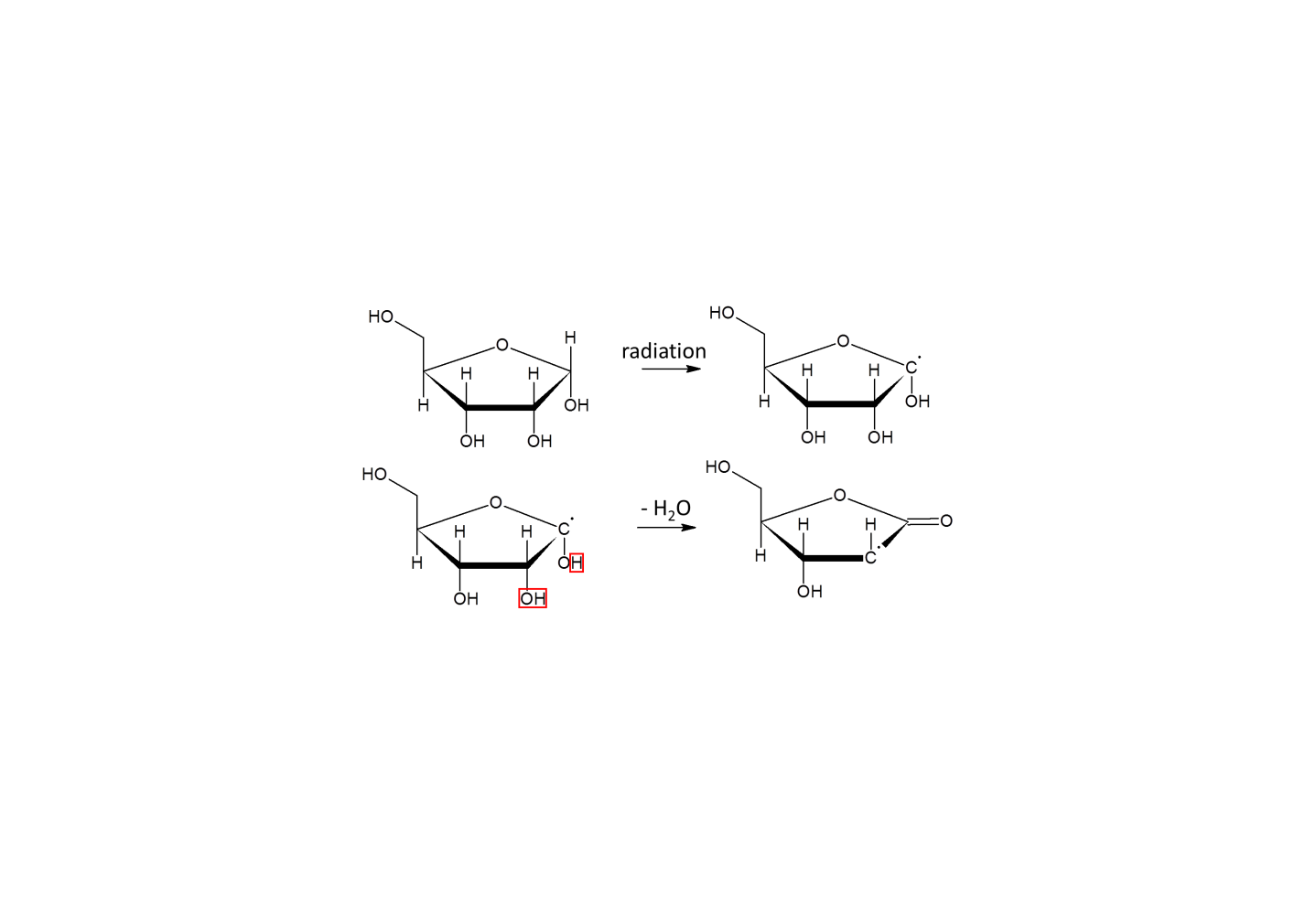


**Figure SI # 9-B.** Degradation of the C1-dehydrogenated radical formed from ribose via water loss. Note, that the same degradation mechanism is not feasible with 2-deoxyribose due to the absence of the 2-OH group.

**SI # 10: B3LYP/6-31+G** optimized geometries used to evaluate the free energy profile of the N-glycosidic bond formation computed at CCSD(T)/6-31+G** level of theory**.

Simplified model of M3/M8 from Figures 1 and SI #8-A

C 1.26374000 -1.30525300 -0.37922300

N 0.54849500 0.03751100 -0.73062000

C 1.65620400 1.00600300 -0.51400900

C 2.58119700 0.33431100 0.27079700

N 2.38680000 -0.97498100 0.46128800

H 0.53755200 -1.98401100 0.07110300

H 1.57596700 -1.70186000 -1.35651400

H 3.43822500 0.82966900 0.72373700

H 1.43910500 2.06085700 -0.58220900

C -0.77113200 0.32407800 0.02206100

O -1.60029100 -0.67359700 -0.49120900

O -1.23504000 1.56104700 -0.44834100

C -0.58240600 0.30199500 1.53047600

H 0.25747100 0.00016800 -1.71039400

C -2.93199800 -0.75419900 0.05157200

H -0.97715500 2.27400800 0.15586600

H -1.50787800 0.62422600 2.01274800

H 0.21622100 0.99188900 1.81285100

H -0.33323300 -0.70221100 1.87696500

H -3.46335200 -1.46225000 -0.58492700

H -3.42819500 0.21936100 0.01327300

H -2.91276300 -1.12876500 1.07918400

Simplified model of M4/M9 from Figures 1 and SI #8-A

C 1.51217700 1.06103100 -0.00011600

N 0.43244400 0.00106600 0.00003800

C 1.09587300 -1.24038600 0.00008700

C 2.45742400 -0.94226700 -0.00001100

N 2.77620000 0.35169900 -0.00012200

H 1.38072500 1.69143100 -0.88998600

H 1.38083900 1.69169200 0.88961600

H 3.23639700 -1.70081200 0.00001600

H 0.56267700 -2.17525800 0.00024100

C -0.85060600 0.26018800 0.00011400

O -1.63873800 -0.83656700 0.00023000

C -1.34720600 1.66693100 0.00013200

C -3.07458000 -0.69622300 -0.00026200

H -2.43422000 1.71772600 0.00075600

H -0.97749700 2.20365200 -0.88354600

H -0.97639100 2.20397200 0.88314800

H -3.45527200 -1.71695000 -0.00057800

H -3.41285600 -0.17380400 -0.89873800

H -3.41350700 -0.17412100 0.89814600

Initital state for the formamidic acid catalysed water elimination step:

6 1.674944 0.685186 -1.250761

7 0.609093 0.515810 -0.133852

6 1.161360 1.408137 0.918022

6 2.487202 1.615429 0.574546

7 2.884985 1.162975 -0.620700

1 1.784155 -0.260012 -1.785297

1 1.236686 1.433792 -1.928136

1 3.195086 2.121279 1.229126

1 0.673760 1.445847 1.879981

6 0.312051 -0.927124 0.290790

8 -0.239632 -1.469170 -0.865669

8 -0.723016 -0.848580 1.257784

6 1.536745 -1.646342 0.831285

1 -0.313041 0.874623 -0.478538

6 -0.554682 -2.875107 -0.843654

1 -0.361195 -0.793321 2.154998

1 1.242279 -2.616267 1.237113

1 1.987782 -1.054448 1.630846

1 2.271051 -1.800056 0.038483

1 -1.126486 -3.058152 -1.753674

1 -1.162898 -3.125523 0.029827

1 0.356584 -3.480130 -0.856010

7 -1.988438 1.615549 -0.857146

6 -3.072550 1.372799 -0.228257

1 -2.137853 2.332709 -1.565716

8 -3.175565 0.473564 0.755783

1 -4.029734 1.862396 -0.416943

1 -2.320379 0.012542 0.930827

Transition state for the formamidic acid catalysed water elimination step:

6 1.515119 0.839917 -1.228544

7 0.561004 0.502943 -0.089612

6 1.114964 1.328061 0.995264

6 2.385633 1.698077 0.610699

7 2.754956 1.336401 -0.638975

1 1.648454 -0.028555 -1.878402

1 1.016280 1.636856 -1.814725

1 0.596531 1.435013 1.936765

6 0.411593 -0.891363 0.172514

8 -0.110529 -1.474857 -0.919601

8 -0.904864 -0.958785 1.295133

6 1.462804 -1.645439 0.952610

1 -1.071099 1.337459 -0.626405

6 -0.184677 -2.918232 -0.993350

1 -0.640609 -0.646394 2.178653

1 1.094580 -2.599324 1.331686

1 1.807360 -1.027504 1.783106

1 2.305769 -1.828643 0.278698

1 -0.727434 -3.124918 -1.914863

1 -0.734966 -3.314563 -0.137331

1 0.815545 -3.353616 -1.043840

1 3.078682 2.244865 1.249139

7 -2.013864 1.731687 -0.796348

6 -3.064761 1.224037 -0.171503

1 -2.137261 2.504766 -1.436857

8 -3.015001 0.266910 0.643856

1 -4.030010 1.690271 -0.399827

1 -1.737388 -0.399436 0.987497

Simplified model of the radical intermediate leading from M4/M9 to M5/M10 from Figures 1 and SI #8-A

6 -1.521753 1.001276 0.007437

7 -0.508375 0.069143 -0.055423

6 -1.125059 -1.179284 -0.057967

6 -2.473939 -0.930033 -0.001872

7 -2.715889 0.430070 0.035970

1 -1.330920 2.063432 0.023889

1 -3.279759 -1.651064 0.015667

1 -0.555257 -2.092555 -0.104862

6 0.869987 0.317982 -0.159643

8 1.583965 -0.761288 0.279339

6 1.372560 1.713385 0.016075

6 2.975623 -0.822220 -0.087085

1 2.427455 1.777987 -0.256921

1 0.823118 2.408060 -0.624194

1 1.270055 2.056137 1.059731

1 3.308754 -1.821828 0.192442

1 3.558084 -0.075235 0.459366

1 3.092082 -0.675756 -1.165324

Simplified model of M5/M10 from Figures 1 and SI #8-A

C -1.36627000 -0.83843400 0.79509500

N -0.52817900 0.04856400 0.18132000

C -1.28449800 0.73362700 -0.75347400

C -2.55526700 0.22384400 -0.64736600

N -2.60059300 -0.75826300 0.32739600

H 1.09884200 -0.39930100 1.35083200

H -1.02080000 -1.50388300 1.57497400

H -3.43437500 0.50711500 -1.20986300

H -0.86458100 1.49415900 -1.39286200

C 0.90398300 0.16559400 0.43095400

O 1.56210200 -0.47847100 -0.66101000

C 1.33890600 1.61779300 0.59957400

H 2.40598800 1.65986300 0.83147500

H 0.79048800 2.07932900 1.42593600

H 1.15876500 2.19016300 -0.31478200

C 2.89187100 -0.91925200 -0.36316900

H 3.23614100 -1.47168300 -1.23906400

H 3.56851000 -0.07635800 -0.18085200

H 2.89325000 -1.58276900 0.51158300

**References**

*43*. Miller, S. L. & Urey, H. C. Organic Compound Synthesis on the Primitive Earth. *Science.* **130**, 245–251 (1959).

*44*. López-Sepulcre, A. *et al*. Shedding light on the formation of the pre-biotic molecule formamide with ASAI. *Mon Not R Astrom Soc.* **449**, 2438-2458 (2015).

*45*. Carota, E., Botta, G., Rotelli, L., Di Mauro, E. & Saladino, R. Current Advances in Prebiotic Chemistry Under Space Condition. *Curr Org Chem.* **19**, 1963–1979 (2015).

*46*. Krocher, O., Elsener, E. & Jacob, E. A model gas study of ammonium formate, methanamide and guanidiniumformate as alternative ammonia precursor compounds for the selective catalytic reduction of nitrogen oxides in diesel exhaust gas. *Appl Catal B-Environ.* **88**, 66-82 (2009).

*47*. Henderson-Sellers, A. W. & Schwartz, A. Chemical evolution and ammonia in the early Earth's atmosphere. *Nature.* **287**, 526-528 (1980).

*48*. Miller, S. L. A production of amino acids under possible primitive earth conditions. *Science. 117*, 528-529 (1953).

*49*. Niether, D., Afanasenkau, D., Dhont, J. K. G. & Wiegand, S. Accumulation of formamide in hydrothermal pores to form prebiotic nucleobases. *Proc Natl Acad Sci USA.* **113**, 4272-4277 (2016).

*50*. Sleep, N. H., Zahnle, K. & Neuhoff, P. S. Initiation of clement surface conditions on the earliest Earth. *Proc Natl Acad Sci USA.* **98**, 3666–3672 (2001).

*51*. Russel, S., *et al*. *Meteoritical Bulletin*, No. 88, 2004 July. *Met Plan Science.* **39**, A217-A272 (2004).

*52*. Clayton, R. & Mayeda, T. *Meteoritical Bulletin*, MB 88 https://www.lpi.usra.edu/meteor/metbull.php?code=17241.

*53*. Frisch, M. J. T. *et al*. *Gaussian 09, Revision A.1*, Gaussian, Inc.: Wallingford CT (2009).
